# Transcriptome Changes in *Hirschfeldia incana* in Response to Lead Exposure

**DOI:** 10.3389/fpls.2015.01231

**Published:** 2016-01-13

**Authors:** Florence Auguy, Mouna Fahr, Patricia Moulin, Mohamed El Mzibri, Abdelaziz Smouni, Abdelkarim Filali-Maltouf, Gilles Béna, Patrick Doumas

**Affiliations:** ^1^Institut de Recherche pour le Développement, UMR DIADE, Equipe RhizogenèseMontpellier, France; ^2^Centre National de l’Energie, des Sciences et des Techniques Nucléaires, Laboratoire de Biotechnologie des Plantes, UBRM-DSVRabat, Morocco; ^3^Institut de Recherche pour le Développement, Laboratoire de Microbiologie et Biologie Moléculaire, Faculté des Sciences, Université Mohammed V-RabatRabat, Morocco; ^4^Laboratoire de Physiologie et Biotechnologie Végétale, Faculté des Sciences, Université Mohammed V-RabatRabat, Morocco; ^5^Laboratoire de Microbiologie et Biologie Moléculaire, Faculté des Sciences, Université Mohammed V-RabatRabat, Morocco; ^6^Institut de Recherche pour le Développement, UMR IPME, Equipe ABIPMontpellier, France; ^7^Institut National de la Recherche Agronomique, UMR Biochimie et Physiologie Moléculaire des PlantesMontpellier, France

**Keywords:** *Hirschfeldia incana*, *Arabidopsis thaliana*, Brassicaceae, functional genomics, lead tolerance, transcriptome

## Abstract

*Hirschfeldia incana*, a pseudometallophyte belonging to the *Brassicaceae* family and widespread in the Mediterranean region, was selected for its ability to grow on soils contaminated by lead (Pb). The global comparison of gene expression using microarrays between a plant susceptible to Pb (*Arabidopsis thaliana*) and a Pb tolerant plant (*H. incana*) enabled the identification of a set of specific genes expressed in response to lead exposure. Three groups of genes were particularly over-represented by the Pb exposure in the biological processes categorized as photosynthesis, cell wall, and metal handling. Each of these gene groups was shown to be directly involved in tolerance or in protection mechanisms to the phytotoxicity associated with Pb. Among these genes, we demonstrated that *MT2b*, a metallothionein gene, was involved in lead accumulation, confirming the important role of metallothioneins in the accumulation and the distribution of Pb in leaves. On the other hand, several genes involved in biosynthesis of ABA were shown to be up-regulated in the roots and shoots of *H. incana* treated with Pb, suggesting that ABA-mediated signaling is a possible mechanism in response to Pb treatment in *H. incana*. This latest finding is an important research direction for future studies.

## Introduction

Heavy metals including Cu, Mn, or Zn, play an essential role in many plant physiological processes but can be toxic if accumulated at high concentrations. Other metals including Cd, As, or Pb, have no biological functions and are extremely toxic even at low concentrations. Lead, a non-essential heavy metal, is widespread in the environment as a result of many human activities including mining and smelting, burning coal, cement manufacture and agricultural practices. It is widely accepted that lead is a serious pollutant, and is toxic not only for plant roots where Pb is taken up, but also when translocated to aboveground part where it can accumulate (Sterckeman et al., 2006). Numerous previous studies have reported a wide range of negative effects following plant exposure to Pb; the visible symptoms of Pb toxicity are rapid inhibition of seed germination, reduced growth and the appearance of chlorosis. In addition, Pb can cause oxidative damage by stimulating the formation of free radicals and reactive oxygen species (ROS), resulting in oxidative stress and DNA damage (Seregin and Ivanov, 2001; Schutzendubel and Polle, 2002). However, some plant species tolerate the presence of Pb and, more interestingly, several species, including *Noccaea caerulescens, Agrostis tennuis*, or *Festuca ovina*, have developed the capacity to accumulate large amounts of lead in their root tissues with limited translocation to the aboveground parts (Vangronsveld et al., 2009). *Hirschfeldia incana*, commonly known as buchanweed, hoary mustard, or Mediterranean mustard, is a medium perennial shrub belonging to the *Brassicaceae* family. It has been identified as a potential hyperaccumulator of various toxic metals including lead (Auguy et al., 2013). Interestingly, *H. incana* was shown to be capable of accumulating more than 3% (dry weight) of lead in its shoots when grown in hydroponic solution containing 100 μM Pb(NO_3_)_2_ without any marked symptoms of toxicity. Such plant species have evolved physiological and molecular mechanisms to enable them to thrive under Pb metal stress. Many studies have already been published on the effects of lead on plants and on their cellular detoxification mechanisms and several molecular players in the Pb homeostatic network have been identified so far. For instance, the tobacco plasma membrane protein NtCBP4 and the *Arabidopsis* gene *CNGC1* have been shown to be components of a transport pathway responsible for Pb entry into plant cells (Sunkar et al., 2000). An *Arabidopsis* P-type ATPase, HMA3, has been reported to improve tolerance by sequestering Pb in vacuoles (Gravot et al., 2004; Morel et al., 2009). It has also been suggested that HMA4, another P-type ATPase, may play a role in Pb efflux in *N. caerulescens* and *H. incana* (Papoyan and Kochian, 2004; Auguy et al., 2013). In *Arabidopsis*, three members of the ABC (ATPase-binding cassette) transporter family AtATM3, AtPDR12, and AtPDR8 contribute to Pb resistance (Lee et al., 2005; Kim et al., 2006, 2007). ACBP1, an acyl-CoA-binding protein, has been reported to be involved in mediating Pb tolerance through accumulation in shoots (Xiao et al., 2008), and *AtMRP3* transcription has been shown to be strongly induced by Pb treatment in *Arabidopsis thaliana* (Zientara et al., 2009). Finally, chelation is an important mechanism controlling heavy metal tolerance involving small molecules such as metallothioneins, phytochelatins and glutathione (Cobbett and Goldsbrough, 2002).

Recent research has focused on identifying the mechanisms that allow organisms to adapt to or alleviate damage caused by metal stress, and in this context, microarray technology is a convenient tool for rapid analysis of plant gene expression patterns under a variety of environmental conditions. The use of cross-species hybridization (CSH), in which the target RNA and microarray probe come from different species, has increased in the last few years. CSH has been used to examine both closely and distantly related species. In the particular case of heavy metals, Becher et al. (2004) hybridized *A. halleri* RNA samples to *A. thaliana* Affymetrix microarrays and analyzed the effect of Zn treatments on gene expression. Similarly, using the same probe-transcript complex, Weber et al. (2006) analyzed the gene profiles after exposure to Cd. In the same line of thought, Hammond et al. (2006) compared Zn gene regulation by hybridizing *N. cearulescens* and *Thlaspi arvense* RNA to *A. thaliana* Affymetrix microarrays. Van de Mortel et al. (2006) examined the performance of CSH in cDNA microarrays of *A. thaliana* with RNA from *N. cearulescens* treated or not with excess Zn. *H. incana*, like *A. thaliana*, belongs to the *Brassicaceae* family. Based on eight cDNA sequences, from 400 to 1100 bp in length, cloned, and sequenced in a previous work, we estimated that *A. thaliana* and *H. incana* shared an average of 89% nucleotide identity within coding regions (Auguy et al., 2013). This level of identity is similar to those obtained between *A. thaliana* and *N. caerulescens* comparison, with on average 88.5% DNA identity in coding regions (Rigola et al., 2006) and 87% DNA identity in the intergenic transcribed spacer regions (Peer et al., 2003). Based on these data, we hypothesized that the use of *Arabidopsis* cDNA microarrays with RNA from *H. incana* should work and would provide information on changes in the transcriptome that occur during plant exposure to Pb. We performed transcriptome analysis of Pb responses in *A. thaliana* and *H. incana* roots and shoots by comparing the responses of a normal plant, *A. thaliana*, and a lead-hyperaccumulating metallophyte, *H. incana* (Auguy et al., 2013). Our aim was to identify any differential responses to Pb that would advance our understanding of metal-tolerance mechanisms. Based on the assumption that the metal responsiveness of putative signal-transduction components points to the functional involvement of the encoded proteins in mediating metal responses, we also hoped to identify candidate genes that could be analyzed further using reverse-genetic approaches.

## Materials and Methods

### Plant Material and RNA Extraction

Four weeks old *H. incana* and *A. thaliana* seedlings were treated in hydroponic cultures with Pb for 3 days, by adding 100 or 40 μM Pb(NO_3_)_2_, respectively, in a fresh BD medium (Broughton and Dilworth, 1971) without phosphate. These two Pb concentrations correspond to approximately 50% of reduction of primary root growth for both species without any visible symptoms (data not shown). In control experiments, BD medium was replaced by fresh BD medium without phosphate.

RNA was extracted separately from the shoots and roots of the control and of the treated *H. incana* and *A. thaliana* seedlings. Total RNA extraction was performed with two different kits depending on the tissue concerned, RNeasy plant mini kit (Qiagen, USA) for roots and SV Total RNA isolation system (Promega, USA) for shoots. A post-treatment with Turbo DNA free (Ambion, USA) removed contaminated DNA. RNA quality was checked and confirmed using Nanodrop 2000 (Thermo Scientific, USA) analysis and non-denaturing gel electrophoresis. Three independent experiments were performed with 8–9 seedlings per treatment and per repetition and used for both microarray analysis and quantitative reverse transcriptase PCR (QRT-PCR) validation.

### Amplification, Labeling, and Hybridization

Imaxio (France), a service provider accredited by Agilent Technologies (USA), performed amplification, labeling, hybridization, data capture, and primary analysis. The Agilent 44K *A. thaliana* microarrays were used for both species. The array, which comprised 43,803 probes, covered the entire *A. thaliana* transcriptome (27 235 cDNA). Two hundred ng of total RNA were used to synthesize cRNA, incorporating the Cy-3 for the control samples and the Cy-5 for the Pb treated samples. After amplification, 825 ng of fragmented cRNA were hybridized to each array at 60°C for 17 h following the manufacturer’s instructions.

### Microarray Data Processing and Analysis

Images were acquired with the Agilent G2505C scanner and treated with the Feature Extraction Agilent software (version 10.7.3). Two normalizations were applied: first, we applied quartile normalization to minimize variance between chips, and second, probe normalization using the median calculated for all the samples. A Gaussian distribution was obtained with the LOG transformation. Only probes for which all replicates for one condition had values higher than the background were retained. Finally, a Student’s *t*-test between treatments was applied with the following parameters: asymptotic *p*-value, *p*-value < 0.05 and Benjamini–Hochberg correction. Microarray data from this article were submitted to the public NCBI Gene Expression Omnibus database (GEO) accession GSE65334 (Edgar et al., 2002). The microarray data is MIAME compliant.

Functional classifications of statistically significant general stress genes were obtained using the web-based Functional Classification SuperViewer (Provart and Zhu, 2003). This classification tool is based on functional information available from the Munich Information Center for Protein Sequences (MIPS) database (Schoof et al., 2004). Class scores were obtained to determine whether certain functional classes were over-represented among statistically significant general stress genes (Provart and Zhu, 2003). Class score means and standard errors were computed on the basis of 100 bootstrap samples of the input gene list, as described in Provart and Zhu (2003). It should be noted that with SuperViewer, genes can belong to more than one classification. Genevestigator software^1^ was used to compare the expression profiles of selected genes with other expression profiles provided in the database.

### QRT-PCR Validation

To validate the microarray results, the expression profile of a subset of differentially regulated genes was checked by qRT-PCR in *H. incana* and *A. thaliana*. Total RNA was extracted in both species in the same way as described for the microarray. Primer sets were designed with Primer 3 software^2^ (**Additional File 1** in Supplementary Material) from conserved gene sequences obtained from the *Arabidopsis* Information Resource database (TAIR^3^). QRT-PCR uses the method already described by Auguy et al. (2013). Dissociation (melting) curves for all the genes were checked for the presence of primer dimers or spurious and not specific products. The correlation coefficients between qRT-PCR and microarray values were calculated.

### Identification of *Arabidopsis mt2b* T-DNA Insertion Mutants

Multiple alignments of nucleotide sequences revealed high sequence identity between *HiMT2b* and A*tMT2b*. In addition, phylogenic trees placed *HiMT2b* as a putative ortholog of *AtMT2b* (unpublished data). Consequently, *Arabidopsis* homozygote plants of T-DNA insertion lines Salk_144899 and Salk_037601 for the gene At5g02380 (*AtMT2b*) were identified by PCR using three different primers. In both T-DNA insertion lines, T-DNA specific primer (LBb1, 5′-GCGTGGACCGCTTGCTCAACT-3′) and two *AtMT2b* specific primers (AtMT2b-F, 5′-GATCCACAACCACAGCTTCC-3′ and AtMT2b-R, 5′-GGACAAAGATCGTTGACAGC-3′) were used. The genotype of the F3 individuals was checked by PCR using gene-specific primers and T-DNA primers. Individual homozygous mutants were backcrossed twice with *Arabidopsis* wild-type Col-0. Root growth and Pb content analysis were performed on *mt2b* mutants and Col-0 wild-type after 13 days culture on media with or without 40 μM Pb(NO_3_)_2_ as previously described (Auguy et al., 2013).

### Lead Quantification

Roots and shoots of both *H. incana* and *A. thaliana* seedlings treated or not with Pb were washed at 4°C twice with 0.2 mM CaSO_4_ and rinsed with cold distilled water. Samples were dried at 72°C for at least 7 days. The dried tissues were subjected to acid hydrolysis and the concentration of lead in the samples was determined by inductively coupled plasma-atomic emission (ICP AES Ultima 2JY, USA) according to the methods previously described (Temminghoff and Houba, 2004; Margui et al., 2007).

## Results and Discussion

### Differences in Pb Content Between *H. incana* and *A. thaliana*

Optimal Pb concentrations for *H. incana* and *A. thaliana* were chosen based on the results we obtained in a previous experiment (Auguy et al., 2013). Pb content was measured in the roots and shoots of *H. incana* and *A. thaliana* cultivated in hydroponic conditions 3 days after treatment with, respectively, 100 and 40 μM Pb (NO_3_)_2_ (**Table 1**). The amount of Pb in roots was of the same order of magnitude in both the two species and showed very high accumulation [65.5 and 82.9 mg Pb/g DW (dry weight), respectively, in *H. incana* and *A. thaliana*]. In contrast, a marked difference in Pb content was measured in the shoots of the two species: 0.346 mg Pb/g DW in *H. incana* and 0.006 mg Pb/g DW in *A. thaliana*. As previously reported [5], Pb transfer from roots to shoots was observed in *H. incana* but not in *A. thaliana* in which almost all Pb only accumulated in the roots. This result confirms that *A. thaliana* has developed a metal exclusion strategy to cope with metal toxicity in the soil by limiting the amount of Pb translocated from the roots to the shoots (Baker, 1981). In contrast, *H. incana* was previously described to tolerate and accumulate Cu, Zn, Tl, and Pb (Poschenrieder et al., 2001; Gisbert et al., 2006; Madejon et al., 2007; Auguy et al., 2013), and indeed, in our experiment, *Hirschfeldia* plants accumulated significantly higher amounts of Pb in aboveground shoots than *A. thaliana* (**Table 1**). This significant difference between the two species confirmed the rightness of our choice of comparing their transcriptome in response to Pb treatment. The response of the non-tolerant and non-accumulating species *A. thaliana* to Pb was considered to be the standard plant response to Pb and was used as a filter to keep only the specific response to Pb in the tolerant Pb accumulating plant *H. incana*.

**Table 1:** Pb content in *Hirschfeldia incana* and *Arabidopsis thaliana* roots and shoots.

	Roots	Shoots
*H. incana*	65.5 ± 6.2	0.346 ± 0.034
*A. thaliana*	82.9 ± 38.6	0.006 ± 0.005


*Pb content was measured 3 days after treatment with 100 and 40 μM de Pb(NO_*3*_)_*2*_, respectively. Data are expressed in Pb mg.g^-*1*^ DW and are the average (±SE) of three independent measurements each made on 5–7 plants.*

### Overview of the Transcriptome Response to Pb Treatment

In order to understand the molecular events underlying the response of *H. incana* to Pb exposure, we measured gene expression in the shoots and roots of *H. incana* plants treated or not with 100 μM of Pb(NO_3_)_2_ for 3 days, using the Agilent 44k *Arabidopsis* array. In parallel, to evaluate differential genes expressed in a Pb tolerant plant versus a non-tolerant plant, we investigated the transcriptome response of *A. thaliana* treated or not with 40 μM of Pb(NO_3_)_2_ for 3 days also using the Agilent 44k *Arabidopsis* array.

In this study, the number of Pb-regulated genes was 2,108 for *H. incana* and 14,800 for *A. thaliana*, with a false detection rate (FDR) < 0.1. **Additional Files 2**–**5** in Supplementary Material list the 20 most Pb-regulated genes in the roots and shoots of *H. incana* and *A. thaliana*. A total of 68.5% of Pb-regulated genes were distributed in the roots and 31.5% in the shoots of *H. incana*, and 93.5% in the roots and 6.5% in the shoots of *A. thaliana* (**Table 2**). The ratio of up- and down-regulated genes in each type of tissue was approximately the same and reached 50%. The marked difference between the number of Pb-regulated genes in *H. incana* and *A. thaliana* could be due to the CHS technique used. Even if *H. incana* and *A. thaliana* are closely related species, it cannot be excluded that some *H. incana* sequences diverged too much from *A. thaliana* sequences and consequently prevented hybridization on the *A. thaliana* arrays. On the other hand, the small number of Pb-regulated genes detected in *Arabidopsis* shoots could be related to the weak concentration of Pb measured in this tissue after 3 days of treatment with 40 μM of Pb(NO_3_)_2_ (**Table 1**).

**Table 2:** Number of regulated genes in roots and shoots of *A. thaliana* and *H. incana* treated with Pb relative to untreated plants.

	Roots		Shoots	
		
	Up-regulated	Down-regulated	Up-regulated	Down-regulated
*H. incana*	795	648	369	296
*A. thaliana*	6737	7095	407	561


**FIGURE 1:**
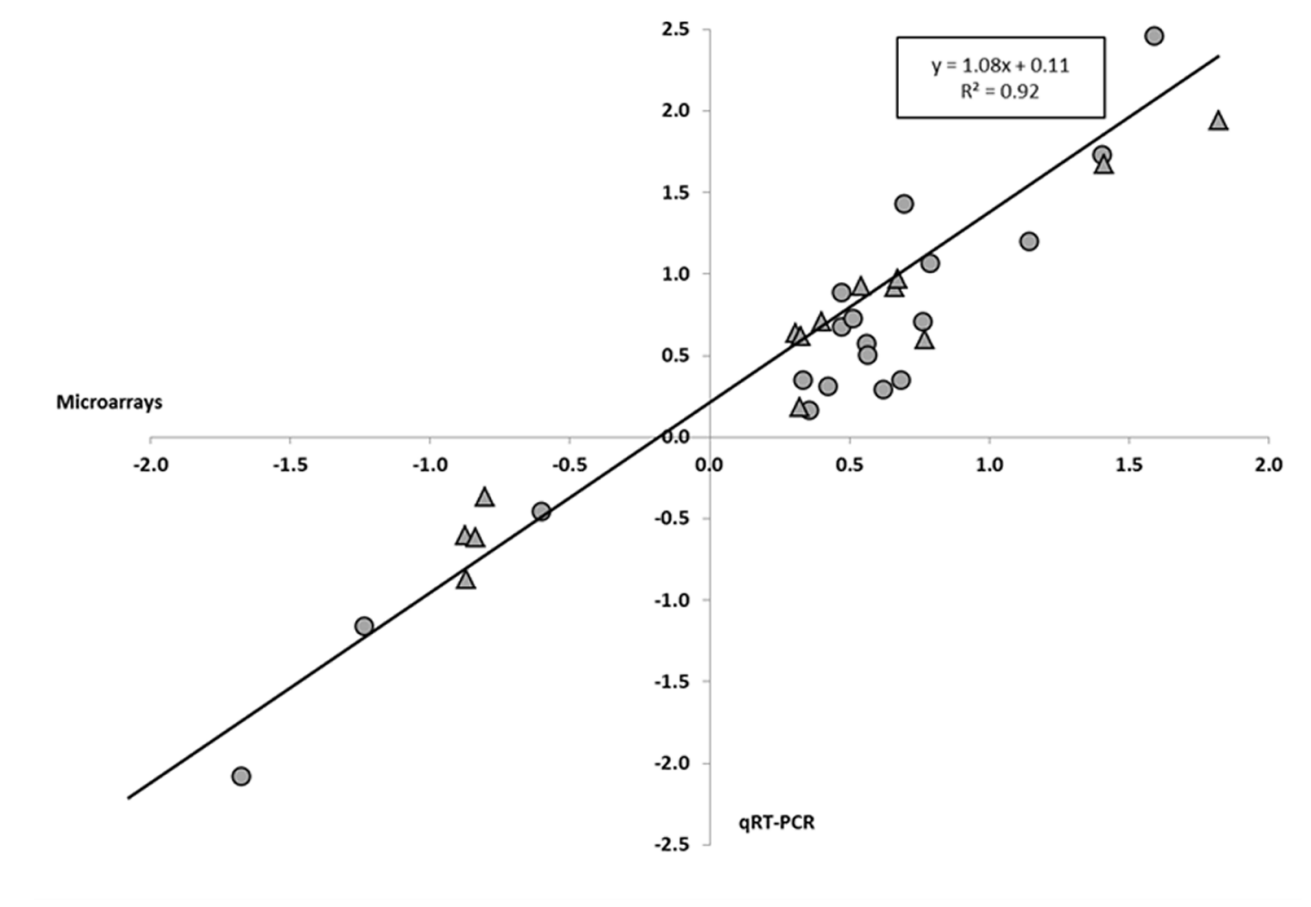
**Pearson’s correlation between gene expression levels determined by quantitative reverse transcriptase PCR (qRT-PCR) and microarrays for *Hirschfeldia incana*.** The diagram represents the correlation between gene expression obtained with the microarray and qRT-PCR for *H. incana* plantlets treated with 100 μM Pb(NO_3_)_2_ for 3 days. Values are Log10 of the fold change (FC) obtained for the same genes with the microarray (*x* axis) and qRT-PCR (*y* axis). FC values for roots (circles) and shoots (triangles) are reported in **Additional File 6** in Supplementary Material.

To validate our microarray results, we performed qRT-PCR to determine the levels of expression of 33 *H. incana* and 6 *A. thaliana* genes randomly selected from the list of genes differentially expressed after Pb treatments. In *H. incana*, the qRT-PCR gene expression results are highly correlated with the microarray gene expression data (**Figure 1**). The coefficient of determination (*r*_2_) between microarray and qRT-PCR data was 0.92, indicating good consistency between the two methods. The genes we tested varied in the same way in the microarray and the qRT-PCR results (**Additional File 6** in Supplementary Material). This clearly validates the transcriptomic results obtained in *H. incana* with Agilent microarray. In *A. thaliana*, in a similar way but with fewer genes, qRT-PCR data were similar to those obtained with the microarray (**Additional File 7** in Supplementary Material), thus validating the *A. thaliana* results obtained with the same microarray.

### Genes Specifically Regulated by Pb Treatment in *H. incana*

**FIGURE 2:**
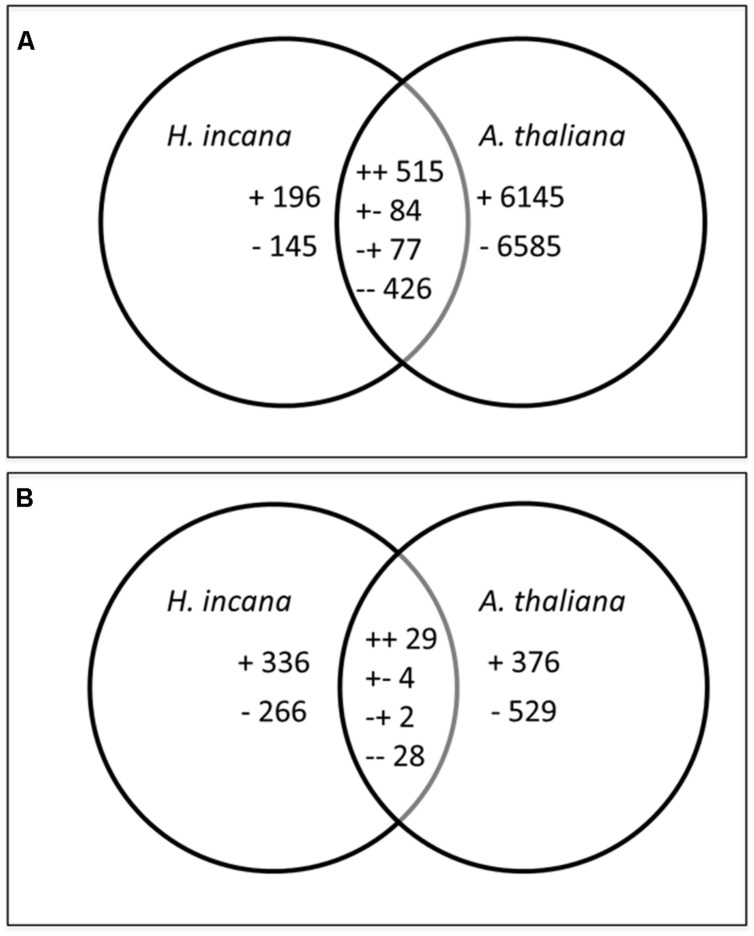
**Venn diagrams representing the distribution of regulated genes between *H. incana* and *Arabidopsis thaliana* in roots **(A)** and shoots **(B)** after Pb exposure.** +, up-regulated; -, down-regulated; ++, up-regulated in both species, --, down-regulated in both species, +-, up-regulated in *H. incana* and down-regulated in *A. thaliana* and -+, down-regulated in *H. incana* and up-regulated in *A. thaliana*.

In order to highlight genes that were specifically regulated by Pb exposure in *H. incana*, we compared its transcriptome with the transcriptome of the Pb-sensitive plant *A. thaliana*. We focused particularly on genes that were specifically Pb-regulated in *H. incana* roots and shoots, (Pb-specific genes), and genes that were regulated in both species (Pb-common genes). These comparisons are illustrated by Venn diagrams (**Figure 2**). The number of Pb-specific genes was higher in shoots than in roots, with 602 and 341 genes, respectively, suggesting a stronger acclimation response by the shoots. Conversely, the number of Pb-common genes was much higher in the roots, with 1,102 Pb-regulated genes in comparison to only 63 genes in the shoots (**Figure 2**). The distribution of up- and down-regulated genes was similar in shoots and roots (55% up-regulated genes). In the Pb-common gene group, we distinguished four categories: genes up-regulated in both roots and shoots, genes down-regulated in both roots and shoots, genes up-regulated in shoots and down-regulated in roots, and genes up-regulated in roots and down-regulated in shoots. The distribution of genes in these four categories was similar in the roots and shoots (**Figure 2**).

In the Pb-specific gene group, only regulated genes with a twofold change (FC) in gene expression were retained. They represented 121genes in roots and 331 in shoots (**Table 3**). For the Pb-common gene category, we introduced another selection step involving a comparison between *H. incana* and *A. thaliana*: only genes differentially expressed in *H. incana* with a FC *H. incana*/FC *A. thaliana* > 2.0 or < 0.5 were retained. This category represented 315 genes in roots and 28 in shoots (**Table 3**). Within this differentially expressed gene category, in the same way as Pb-specific genes, only regulated genes (those with a FC > 2.0 or FC < 0.5) were selected. After this second step, the Pb-common gene list included 227 genes in roots and 26 in shoots (**Table 3**). Finally, including specific and common genes, the selected gene lists comprised 348 genes in roots and 356 genes for roots and in shoots (**Table 3**, **Additional Files 8** and **9** in Supplementary Material). Among these, only 42 genes were regulated both in roots and shoots (in bold in **Additional Files 8** and **9** in Supplementary Material). As mentioned in the introduction, several genes were already known to be involved in plant tolerant response to Pb exposure including *ACBP1*, *AtATM3*, *NtCBP4*, *CNGC1*, *HMA3*, *HMA4*, *AtMRP3*, *AtPDR8*, and *AtPDR12*. Contrary to the data in the literature, no hybridization signal was measurable for *ACPB1*, *MRP3*, *PRD8*, and *PRD12* and a FC less than 2 or 0.5 was measurable for *ATM3, CBP4, CNGC1*, *HMA3*, and *HMA4*. This particular focus on these Pb-specific genes illustrates the limits of the DNA CSH technique.

**Table 3:** Selection steps applied to highlight *H. incana* candidate genes regulated in response to Pb exposure in roots and shoots.

Category	Treatment	Roots		Shoots	
Specific genes	Total	341		601	
	FC > 2.0 or FC < 0.5	62 Up-reg	59 Down-reg	192 Up-reg	138 Down-reg
Common genes	Total	1102		63	
	FC Hi/FC At > 2.0 or < 0.5	315		28	
	FC > 2.0 or FC < 0.5	125 Up-reg	102 Down-reg	18 Up-reg	8 Down-reg
Candidate genes		187 Up-reg	161 Down-reg	210 Up-reg	146 Down-reg


**H. incana* specific genes were directly chosen on the basis of their fold change (FC) ratio (FC > 2.0 or FC < 0.5). *H. incana* common genes were first selected on the basis of their FC related to those from *A. thaliana* (FC Hi/FC At > 2.0 or < 0.5) and then FC on the basis of the FC ratio (FC > 2.0 or FC < 0.5). (Fold Change, FC; Hi, *H. incana*; At, *A. thaliana*; down-reg, down-regulated genes; up-reg, up-regulated genes).*

### Functional Classification of Pb-Responsive Genes

**FIGURE 3:**
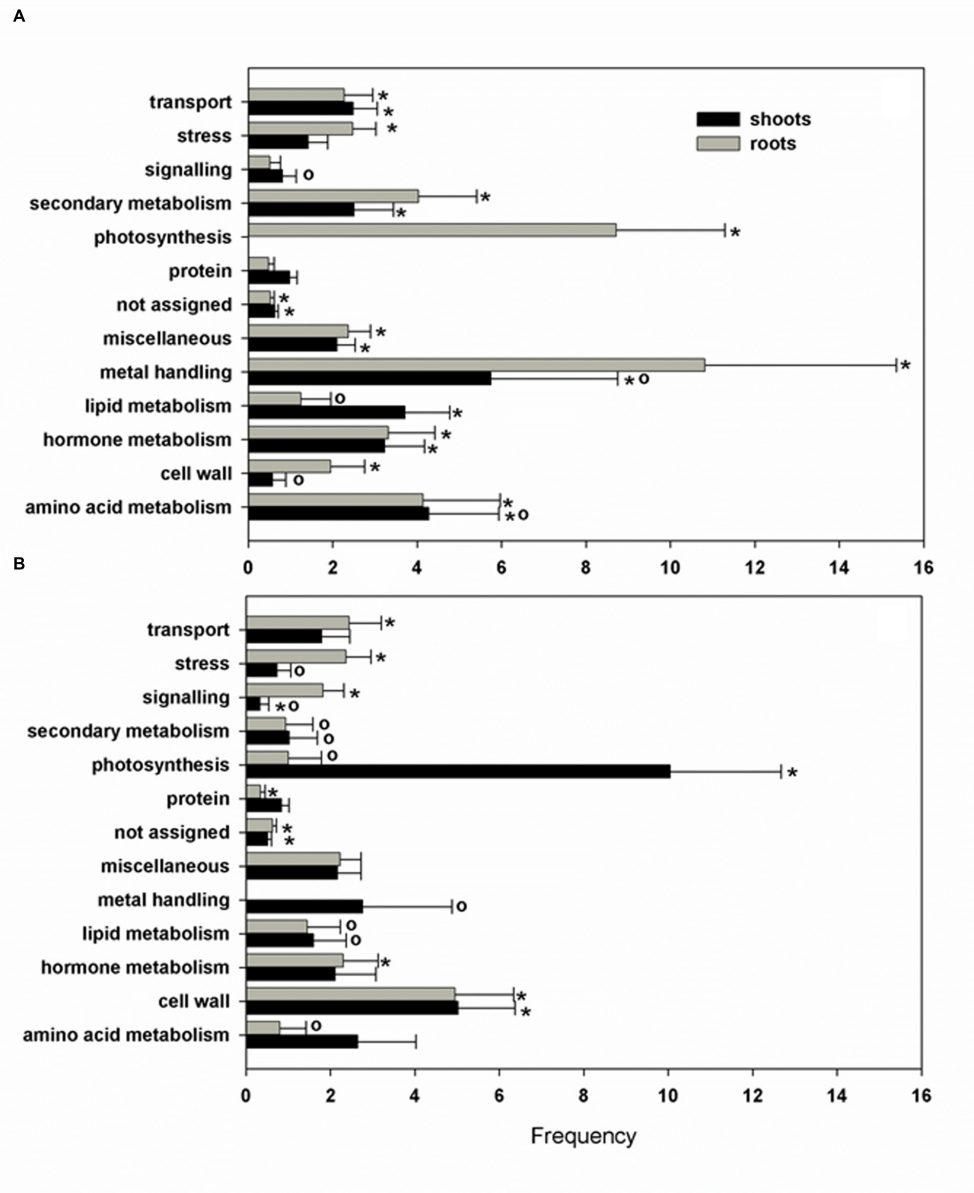
**Biological process classification of Pb responsive selected transcripts.** The gene ontology was obtained using the Classification SuperViewer software with MapMan classification source for selected up- **(A)** and down-regulated **(B)** genes. Normed frequency was calculated as follows: (Number in class input set/Number in classified input set)/(Number in class reference set/Number in classified reference set). ^∗^Significant class (*p* > 0.05), o, size less than 5 genes.

To evaluate the functional significance of the differentially expressed genes in response to Pb exposure in *H. incana*, the biological processes with over-represented regulated genes were identified in our analysis using Classification SuperViewer tools (Provart and Zhu, 2003) available at the Bio-Array Resource for Plant Functional Genomics (BAR) website with MapMan classification source. Several biological processes, categorized as photosynthesis, cell wall, and metal handling, were particularly enriched by Pb exposure in both roots and shoots (**Figure 3**).

Photosynthesis is considered to be one of the most sensitive metabolic processes to Pb toxicity (Singh et al., 1997). Lead toxicity has multifunctional adverse effects on photosynthetic CO_2_ fixation and reduces the level of photosynthetic pigments, alters chloroplast ultra-structure and reduces the enzymatic activity of CO_2_ assimilation (Parys et al., 1998; Islam et al., 2008; Belatik et al., 2013). In the present study, several genes directly involved in photosystem II encoding, respectively, a light-harvesting chlorophyll-a/b (Lhc) protein (At2g05070), a Mog1/PsbP/DUF1795-like protein (At3g56650), a subunit of the NAD(P)H dehydrogenase complex (At1g14150), a PsbP-like protein 1 (At3g55330) and a photosystem II reaction center subunit W (At2g30570) were all down-regulated in shoots (**Table 4**). Three genes involved in the Calvin cycle (At3g12780, At1g56190, and At1g73110) encoding phosphoglycerate kinase proteins were also down-regulated. Pb may play a role similar to that of Mg, which at high concentrations non-competitively inhibits phosphoglycerate kinase activity (Larsson-Raźnikiewicz, 1967). Interestingly, increased expression of photosynthetic genes was observed in roots, with the exception of At1g10960 encoding a ferredoxin 1 for which the gene expression was down regulated by 0.4 (**Table 5**). Because roots are thought to be a non-photosynthetic organ, the biological relevance of this phenomenon has received little attention but the majority of the genes affected (8/11) are members of Lhc family of photosynthesis-associated nuclear genes (At1g29910, At1g29930, At2g34420, At2g34430, At3g47470, At3g54890, At5g28450, and At5g54270) and can be expressed in specialized territories, including the root tip (Sawchuk et al., 2008). The putative interactions between Lhc gene expression, root localization and Pb exposure are somewhat unclear but what is particularly interesting is that abscisic acid (ABA) was required for full expression of different Lhc members and physiologically high levels of ABA enhanced Lhc expression (Liu et al., 2013).

**Table 4:** GO enrichment analysis of differentially expressed genes in shoots.

Functional category	AGI	FC	Description
Photosynthesis	At1g56190	0.499	Phosphoglycerate kinase family protein
	At2g30570	0.427	Photosystem II reaction center W
	At3g55330	0.418	psbP-like protein 1
	At3g12780	0.413	Phosphoglycerate kinase 1
	At1g11860	0.372	Glycine cleavage T-protein family
	At1g14150	0.356	psbQ-like protein 2
	At1g73110	0.299	*p*-loop containing nucleoside triphosphate hydrolases
	At3g56650	0.266	Photosystem II reaction center PsbP family protein
	At2g05070	0.249	Light harvesting complex 2.2
Cell wall	At4g17030	16.664	Expansin-like B1
	At3g48530	2.564	Related protein kinase regulatory subunit gamma 1
	At5g16190	0.470	Cellulose synthase like A11
	At5g26670	0.387	Pectinacetylesterase family protein
	At1g70370	0.362	Polygalacturonase 2
	At1g04680	0.356	Pectin lyase-like superfamily protein
	At1g03870	0.302	Arabinogalactan 9
	At4g03210	0.301	Xyloglucan endotransglucosylase/hydrolase 9
	At1g23480	0.266	Cellulose synthase-like A3
	At3g10720	0.239	Plant invertase/pectin methylesterase inhibitor superfamily
	At3g23730	0.224	Xyloglucan endotransglucosylase/hydrolase 16
	At2g06850	0.212	Xyloglucan endotransglucosylase/hydrolase 4
	At5g04970	0.196	Plant invertase/pectin methylesterase inhibitor superfamily
	At4g16980	0.103	Arabinogalactan-protein family
Metal handling	At3g09390	4.577	Metallothionein 2A
	At5g01600	2.514	Ferretin 1
	At5g02380	2.415	Metallothionein 2B
	At3g24450	0.412	Heavy metal transport/detoxification superfamily protein


**Table 5:** GO enrichment analysis of differentially expressed genes in roots.

Functional category	AGI	FC	Description
Photosynthesis	At5g54270	7.424	Chlorophyll B-binding protein 3
	At1g29910	7.103	Chlorophyll A/B binding protein 3
	At1g29930	6.547	Chlorophyll A/B binding protein 1
	At3g54890	5.506	Photosystem I light harvesting complex gene 1
	At5g28450	5.442	Chlorophyll A-B binding family protein
	At2g34430	5.218	Light harvesting complex gene B1B1
	At2g34420	5.141	Light harvesting complex gene B1B2
	At3g47470	5.022	Chlorophyll-protein complex I subunit A4
	At1g51400	4.379	Photosystem II 5 kD protein
	At2g30570	4.202	Photosystem II reaction center W
	At1g10960	0.414	Ferredoxin 1
Cell wall	At4g17030	25.232	Expansin B1
	At5g06860	4.580	Polygalacturonase inhibiting protein 1
	At5g17420	2.420	Cellulose synthase family protein
	At3g27400	2.197	Pectin lyase-like superfamily protein
	At1g67070	2.126	Mannose-6-phosphate isomerase, type I
	At2g37090	2.039	Nucleotide-diphospho-sugar transferase superfamily
	At1g26240	0.458	Proline-rich extensin-like family protein
	At2g28950	0.456	Expansin A6
	At1g57590	0.445	Pectinacetylesterase family protein
	At4g03210	0.423	Xyloglucan endotransglucosylase/hydrolase 9
	At3g15370	0.416	Expansin A12
	At4g08410	0.406	Proline-rich extensin-like family protein
	At5g53250	0.395	Arabinogalactan protein 22
	At5g45280	0.310	Pectinacetylesterase family protein
	At4g01220	0.250	Nucleotide-diphospho-sugar transferase family protein
	At4g01630	0.131	Expansin A17
	At4g30280	0.130	Xyloglucan endotransglucosylase/hydrolase 18
	At3g10720	0.120	Plant invertase/pectin methylesterase inhibitor superfamily
	At1g21310	0.090	Extensin 3
Metal handling	At4g08570	13.918	Heavy metal transport/detoxification superfamily protein
	At1g22990	5.758	Heavy metal transport/detoxification superfamily protein
	At2g28660	6.161	Chloroplast-targeted copper chaperone protein
	At4g39700	2.625	Heavy metal transport/detoxification superfamily protein
	At5g17450	2.765	Heavy metal transport/detoxification superfamily protein


The second biological process group particularly enriched by Pb exposure contained genes encoding cell wall proteins in both shoots and roots (**Tables 4** and **5**). Recent studies in different plant species have shown that the plant cell wall could be modified in response to Pb (Krzeslowska, 2011). Modifications of the cell wall mainly concern an increase in the amount of polysaccharides, in particular pectins. In the present study, Pb treatment induced a reduction in xyloglucan endotransglucosylase gene expression (At2g06850, At2g23270, and At4g03210) and could play an important role in Pb-induced root growth inhibition by causing cell wall modification and reduction of cell elongation. Similar observations have been reported for Al toxicity in *Arabidopsis* (Yang et al., 2011).

**FIGURE 4:**
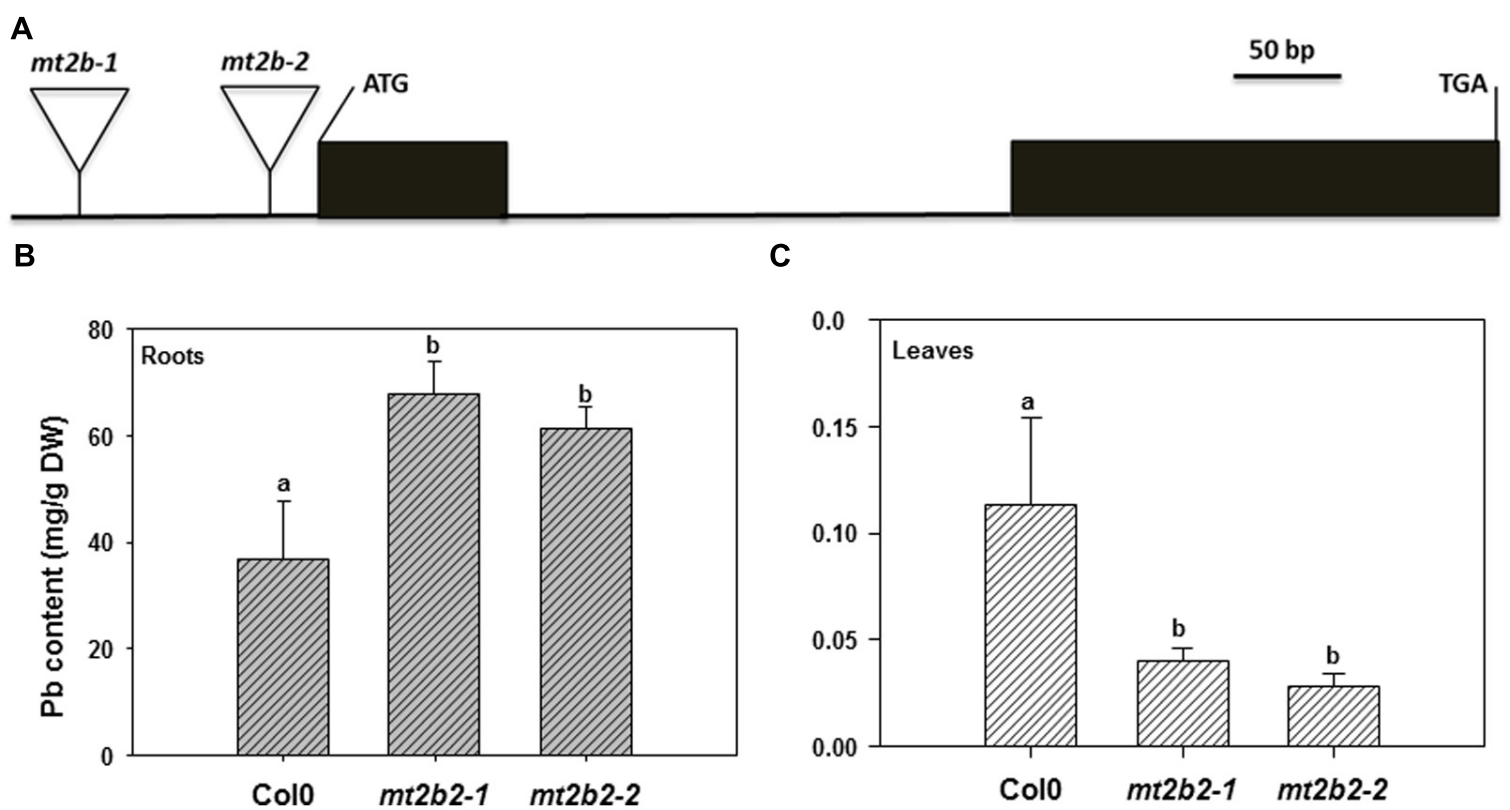
**Functional characterizations of the *Mt2b* gene.** **(A)** Intron-exon organization of the *Arabidopsis Mt2b* gene (AT5G02380) and T-DNA locations. Solid black boxes and the solid line indicate coding regions and introns, respectively. The position of the 2 T-DNA insertions is indicated by a triangle. **(B)** and **(C)** Lead accumulation in roots and shoots of, respectively, wild type ecotype Columbia (Col0) and two mt2b mutant line seedlings of *A. thaliana*. Seedlings were collected after 13 days culture on media with or without 40 μM Pb(NO_3_)_2_. All results are the average value (±SE) of three independent replicates. The letters represent homogenous subgroups of roots and shoots, using LSD *post hoc* test at an α = 0.01 significance level.

The last biological group enriched by Pb exposure that emerged from this analysis encompasses the group of genes involved in metal handling. The frequency of up-regulated genes in roots was greater than 10, i.e., it was the most enriched category of the data (**Figure 3A**). Among the genes associated with this category, four belonged to the metallochaperone-like protein family (At4g39700, At5g17450, At1g22990, and At4g08570). Expression of these genes was increased by Pb-exposure with a fold-change ranging from 2.6 to 13.9 (**Table 5**). The function of these metalloproteins is unknown, but two of them (At1g22990 and At4g08570) are known to be involved in Cd tolerance (Tehseen et al., 2010). Another gene in the metal handling category, At2g28660, encodes putative soluble proteins with a heavy metal binding domain like Cys-x-x-Cys and a putative chloroplast targeting sequence is associated with the Cu chaperone family (Abdel-Ghanya et al., 2005). The expression of this gene was up regulated 6.2-fold in *H. incana* roots treated with Pb compared to the control. This protein possesses a heavy metal fixation domain and could act as a putative Pb fixation site. Only three genes up-regulated in shoots were identified in the metal handling category (**Table 4**) and two genes code for metallothioneins *MT2a* (At3g09390) and *MT2b* (At5g02380). Expression levels of these two genes were, respectively 4.5 and 2.4 in shoots treated with Pb and in the control (**Table 4**). Although the protective role of metallothioneins against Cd is well known in mammals (Klaassen et al., 1999), their role in plant tolerance to heavy metals is less well known. MT2a is known to be localized the cytosol and could chelate heavy metals, but is not involved in vacuolar sequestration Lee et al., 2004). In a previous study, we demonstrated the role of *MT2a* in Pb tolerance using *Arabidopsis* T-DNA insertional mutants (Auguy et al., 2013). Treatment with Pb highly significantly reduced primary root length in *mt2a* (48%) mutants. This reduction in primary root growth reflected increased sensitivity to Pb in *Arabidopsis* T-DNA insertion mutants and suggests that the *MT2a* gene is involved in lead tolerance. No effect of the mutation was observed on the Pb content in shoots and in roots of mt2a-mutant compared to wild-type plants (Auguy et al., 2013). Another metallothionein gene identified in the present study was *MT2b* (At5g02380). *MT2b* was previously described as being expressed in the phloem of all organs (Guo et al., 2008) and involved in Cu tolerance in *Silene vulgaris* or in Cd tolerance in Tobacco (Van Hoof et al., 2001; Grispen et al., 2011). In order to examine the functions of *MT2b* gene in Pb tolerance in *Arabidopsis*, we used a reverse-genetic approach with two independent *Arabidopsis* T-DNA insertion lines (Salk_144899 and Salk_037601) for the *MT2b* gene obtained from the Salk institute (**Figure 4A**). We measured the Pb content in roots and shoots of the 2 week-old wild-type and mt2b-mutants. Roots of both mutant lines contained 1.5-fold more Pb than the roots of wild-type plants (**Figure 4B**) and shoots of both mutant lines contained 3.3-fold less than those of wild-type plants (**Figure 4C**). No effect of the mutations was observed on the primary root growth of *mt2b* mutants compared to the wild-type plants (data not shown). These results suggest a role of MT2b in Pb accumulation through a perturbation of root-to-shoot translocation by reducing Pb content in leaves and increasing in roots. Very recently, using a *MT* quadruple mutant (*mt1a/mt2a/mt2b/mt3*), Benatti et al. (2014) showed that *Arabidopsis* metallothioneins are important for the accumulation and distribution of Cu in leaves and seeds, confirming the role of MT in the accumulation of metals in plants.

### Comparison of Profiles with Other Transcriptomic Experiments

**FIGURE 5:**
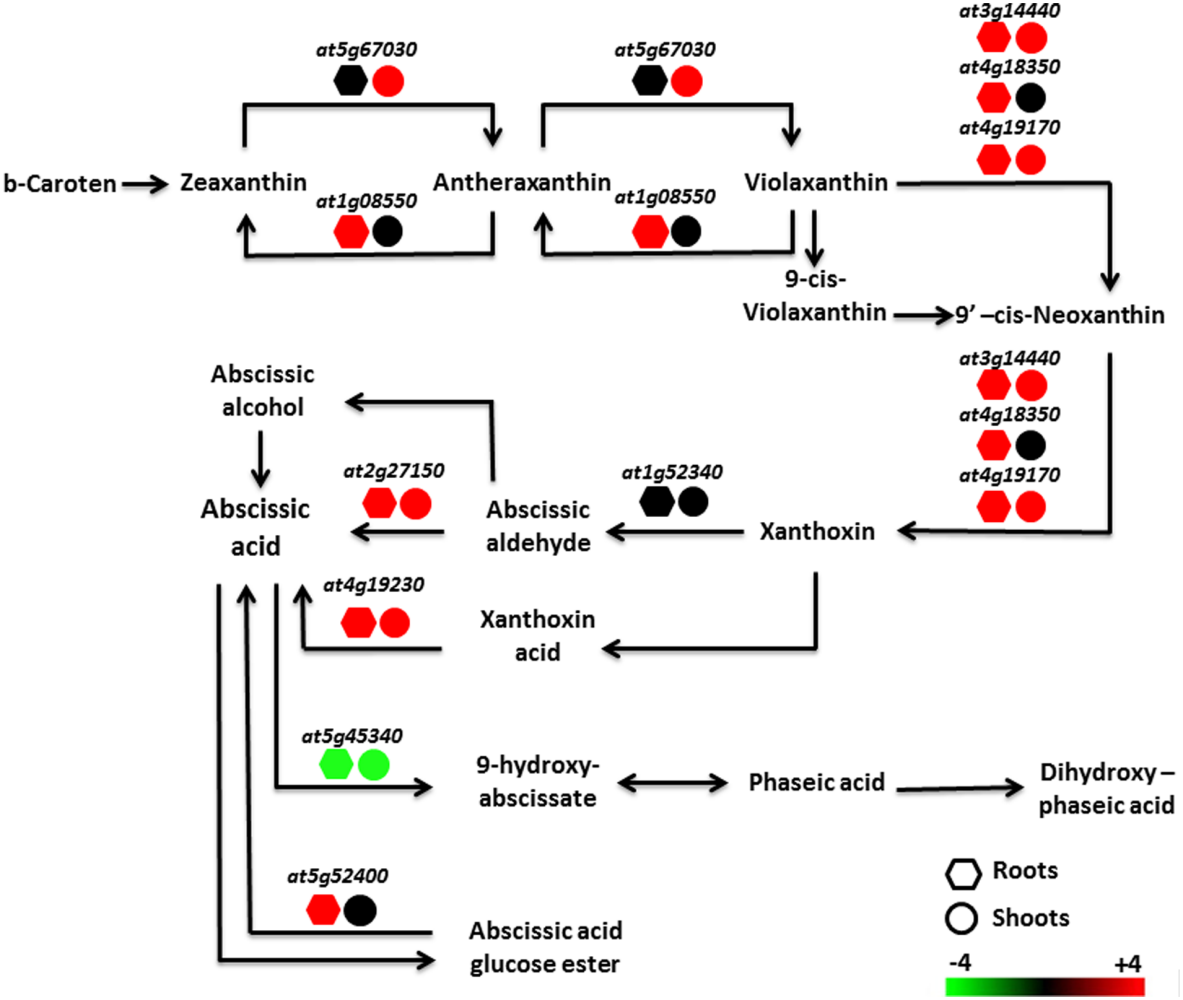
**Regulation of the abscisic acid metabolism pathway in *H. incana* roots and shoots treated with Pb.** Red and green represent, respectively, an increase and a decrease in regulation, compared with untreated plants. Expression value scale is provided.

Data were analyzed using Genevestigator software (Nebion AG, Switzerland) with the Genevestigator tool “signature^4^”. Selected genes in both roots (348 genes) and shoots (356 genes) that responded specifically to Pb exposure in the Pb-tolerant *H. incana* were compared to the Genevestigator “Perturbation” database (**Tables 6** and **7**, respectively). A comparison of the complete profiles for roots and shoots is given in **Additional Files 10** and 11 in Supplementary Material, respectively. In the Perturbation database, experiments with the most significant similarity to our Pb experiment include data from abiotic stress-related studies, mainly salt stress-related, osmotic-related and drought experiments as well as abscisic acid (ABA) study (**Tables 6** and **7**). Furthermore, it is important to note that, from the biological process classification of Pb responsive selected transcripts (**Figure 3**), the gene composition of the hormone metabolism class underline the over-representation of ABA subclass (**Additional File 12** in Supplementary Material). To confirm this hypothesis, total expression data from our Pb experiment were re-analyzed. Several genes involved in the biosynthesis of ABA were shown to be up-regulated in *H. incana* roots and shoots treated with lead (**Figure 5**), suggesting induction of ABA biosynthesis when *H. incana* plants were exposed to Pb treatment. Interestingly, even the Atg52400 gene involved in the ABA catabolism pathway, via phaseic acid (PA), was largely down-regulated, also leading to potential ABA accumulation. Abscisic acid, as a stress signal, is known to enhance plant tolerance to several environmental stresses, including low temperature, salt, drought, and heavy metals (Bellaire et al., 2000; Verslues and Zhu, 2005; Hong et al., 2013; Li et al., 2014). The increase in tolerance is partly due to the enhancement of the antioxidant defense system, which prevents the accumulation of ROS (Bellaire et al., 2000; Jiang and Zhang, 2002). On the other hand, in plants in contact with Pb, transpiration is decreased (Barcelo and Poschenrieder, 1990). This decrease in transpiration could be due to a reduction in growth, or at least to a reduction in leaf surface area and/or to the fact that when Pb is fixed to the cell wall it reduces plasticity and eventually disturbs the osmotic balance. Accumulation of Pb in the cell leads to accumulation of ABA in the shoots, results in stomata closure and reduces loss of water through transpiration (Parys et al., 1998; Pourrut et al., 2011). Taken together, these data indicate an intricate relationship between plant tolerance to Pb and ABA pathways. Recently a similar conclusion was reached by Shukla et al. (2014), who suggested that ABA-mediated signaling could be a major mechanism in response to metal gold exposure in *Arabidopsis*.

**Table 6:** Top 10 most similar profiles for *H. incana* roots from perturbation category (Genevestigator database).

Sample identification from Genevestigator	Relative similarity
Osmotic study 2 (late)/untreated root (late)	1.238
Salt study 5 (cpc-1 try-82)/mock treated primary root tip (cpc-1 try-82)	1.164
Salt study 5 (wer-1 myb23-1)/mock treated primary root tip (wer-1 myb23-1)	1.158
Salt study 5 (col-0)/mock treated primary root tip (col-0)	1.150
Osmotic study 2 (early)/untreated root (early)	1.149
Salt study 5 (scm-2)/mock treated primary root tip (scm-2)	1.148
Dicamba herbicide (10 h)/H2O treated seedling (10 h)	1.114
ABA study 6 (srk2cf)/untreated plant (srk2cf)	1.112
Salt study 2 (early)/untreated root (early)	1.111
ABA (3 h)/mock treated seedling (3 h)	1.111


*Relative Similarity indicates the degree of their resemblance: the higher value the higher similarity relative to the average similarity.*

**Table 7:** Top 10 most similar profiles for *H. incana* shoots from perturbation category (Genevestigator database).

Sample identification from Genevestigator	Relative similarity
Osmotic (late)/untreated green tissue (late)	1.468
Drought study 2 (Trans.)/untreated leaf (Trans.)	1.414
Drought study 11 (hai1-2)/mock treated seedling (hai1-2)	1.412
Salt (late)/untreated green tissue (late)	1.411
Salt study 3 (atmy44)/H2O treated rosette leaf (atmy44)	1.399
ABA study (srk2cf)/untreated plant (srk2cf)	1.386
ABA study (col-0)/untreated plant (col-0)	1.366
Drought (wt)/untreated leaf (col)	1.362
Drought study 11 (col-0)/mock treated seedling (col-0)	1.354
Osmotic study 4 (shoot)/mock treated col-0 shoot	1.347


*Relative Similarity indicates the degree of their resemblance: the higher value the higher similarity relative to the average similarity.*

## Conclusion

The comparative transcriptomic analysis between the Pb-hyperaccumulating and Pb-tolerant plant *H. incana* and the Pb-sensible model plant *A. thaliana* identified a set of genes differentially expressed in response to Pb exposure. These genes were particularly over-represented in the photosynthesis, cell wall structure, and metal handling biological processes. In addition several genes involved in the ABA biosynthetic pathway were up regulated in response to lead exposure suggesting that ABA-mediated signaling is involved in plant response to lead. In addition *H. incana* could be considered as a good experimental model to identify genes involved in lead tolerance and accumulation in plants.

## Availability of Supporting Data

The data discussed in this publication have been deposited in NCBI’s GEO (Edgar et al., 2002) and are accessible through GEO Series accession number GSE65334 (http://www.ncbi.nlm.nih.gov/geo/query/acc.cgi?acc=GSE65334).

## Author Contributions

AF and PD conceived and designed the experiment. AF, MF, and PM collected the experimental data. ME, AF-M, and GB contributed reagents/materials/analysis tools. AF, AS, and PD analyzed data. AF and PD wrote the manuscript. GB assisted with the interpretation of the results and provided editorial support for the manuscript. All authors have read, edited, and approved the current version of the manuscript.

## Conflict of Interest Statement

The authors declare that the research was conducted in the absence of any commercial or financial relationships that could be construed as a potential conflict of interest.

## Footnotes

1 https://genevestigator.com/gv/

2 http://primer3.wi.mit.edu/

3 http://www.arabidopsis.org/

4 https://www.genevestigator.com/gv/doc/signature.jsp
