# Supplementary material for: Transcriptome Changes in Hirschfeldia incana in Response to Lead Exposure
Source: Front Plant Sci. 2016 Jan 13;6:1231. doi: 10.3389/fpls.2015.01231 (PMC4710698; doi:10.3389/fpls.2015.01231)
Supplement: Supplementary file 1 [file Data_Sheet_1.PDF]

Additional file 1.
Primers used for the qRT-PCR validation.

ID	Forward Primer (5'-3')	Reverse Primer (5'-3')
AT1G06650	TCCTCATGTTTCCGGCGCTCT	ACATGCGACAGACACTCGAGCT
AT1G15180	TACGCAGCAATGTCTCTTGC	GGTTTCCTTGTCACTGCTGA
AT1G22990	CAAAGGAGCAAAATCAGTGGA	AGCCACCATTGTGTATGGAA
AT2G26020	CATCACCTTTATCTACGCTGCTC	TTGGTACTTCAAAAGCTGCAAA
AT2G28660	TCCACTGATGACCAGGTTGT	TATGACGTCACCCCTTCCAT
AT3G12750	TGCATCTGCTGGGATACTCA	CAGCCAAGTGAAGCCAGAG
AT4G08570	ACAAGAAGGCACCACCAAAC	CGCAAGAATTAGGGTTCTCG
AT4G13420	CAAAGCACTTGCGATTCCTA	AGGTCATGCCAACCTTGAGA
AT4G17030	GGAAGAGATATCAATAACGGTGAAG	TCCATAGTCGCCATGAAACA
AT4G25100	CCCTTGTGCTCGGCTCTTTCCC	GCTTCCCAAGACACAAGATTGGTCA
AT4G35090	ACCGTACCTTTACACCAGAGAGGCA	ATCCAGATACTGCGGATTTCATGCG
AT5G01600	CAACGTTGCTATGAAGGGACT	TTCCTCCTCTTTGGTTCTGG
AT5G02380	GCAAGTGCAACCCTTGTACC	CCGGAAAAAATCAGCCAATTA
AT5G06530	TAACCCATTTTCCACACGAA	TCATTGTCAAGGATTGGTCGT
AT5G09930	TCAAAGATGTCAAAGGCTGAGA	CTTGGCATTTTTGCTGGACT
AT5G11930	ACATCAGAGTCAAAGATCGGACGGC	GAAGCTTCTTCATGACGTGGCACA
AT5G13580	GGGGCTTCTTCTTTCGAATC	TTTCAAACCAGTCCTCCATAAGA
AT5G15410	CGGTTACTTCAGTATGCAGCTATG	AGATGATCATGCGGTCGAA
AT5G38960	CCCGAATGTTCTTGCAAAGG	GAATGATCACCTTAGGATCCAACT
AT1G74670	GGCAACAAACAAGTGTGTCC	TCCACCTTGTTGAGTCTTCCA
AT1G77510	AAAGATCAAAGTCTTGCCTTCC	TGATGATGAGGGGAACTTGG
AT2G36380	TCAAAAATGGATTCGGGTTC	GGACAACGGCTACAACTGGT
AT2G45180	CGCCTCTTAAAGCCAATGTC	GGTTAGATCAATAGGAACCTTGAG
AT3G09390	TCTCCGGCGAGACAACCACAA	TTCTCAGCGTTGTTACTCTCCCCT
AT3G50560	AGACCGGAGAGCTTGGACAC	TTGGGTTTGATGGTCGGATA
AT3G59140	GAAGGATGAGAACTCTTTGTTCG	TGAGACCAATACTCTTTCACAAGC
AT4G11600	AGATTCTTGCGTTTCCGTGT	CGGGTACTCAGCCTTGAAAC
AT4G33020	TACATGGCGCTTGTGGATT	TCAACACTCATCTTCTTGCTCA
AT5G18600	AGATCGAGCAGGCGTTGTT	CGCTTAAGCATGGGAATCAA
AT5G45690	TGGCTGATTATTGGGTTCATC	TTTCGATTATGTCAACCGCTAA
AT5G59330	TTGGCTCTCAGGTTCTTCAT	GAAACTATGCACACCGTCAAAA

Additional file 2.

List of the 20 most up- and down-regulated genes from *H. incana* roots after 3 days of Pb exposure (100 µM $Pb(NO_3)_2$ in hydroponic culture). The fold change control versus treated was validated by a Student test ($P<0.01$).

Probe Name	FC	AGI	Description
A_84_P239215	50.67	AT2G26010	Putative plant defensin 1.3 (AtPDF1.3)
A_84_P310613	39.04	AT2G26020	Putative plant defensin 1.2b (AtPDF 1.2b)
A_84_P825792	25.23	AT4G17030	Putative expansin (AtEXPR1)
A_84_P18335	23.99	AT3G02480	Putative LEA-type (group LEA_4) protein of unknown function
A_84_P856766	20.02	AT3G09950	Protein of unknown function
A_84_P18843	19.42	AT5G66400	Putative LEA-type (group dehydrin) protein of unknown function
A_84_P21673	18.05	AT1G52400	Putative beta-glycosyl hydrolase (AtBGLU18/AtBG1)
A_84_P17268	16.63	AT2G14610	Pathogenesis-related protein. activated during pathogen response (AtPR1)
A_84_P194044	16.55	AT5G07330	Putative membrane protein of unknown function
A_84_P605408	14.18	AT4G12580	Protein of unknown function
A_84_P23247	13.92	AT4G08570	Protein of unknown function. contains metal ion-binding HMA-type domain
A_84_P12638	13.32	AT3G08860	Putative alanine-glyoxylate aminotransferase (AtPYD4)
A_84_P14899	13.23	AT5G10930	Putative SNF1-related protein kinase (AtSnRK3.24/AtPKS19/AtCIPK5)
A_84_P20704	11.67	AT5G59320	Lipid transfer protein (AtLTP3)
A_84_P868627	11.34	AT3G21370	Putative beta-glycosyl hydrolase (AtBGLU19)
A_84_P16611	10.37	AT4G02280	Sucrose synthase/sucrose-UDP glucosyltransferase (AtSUS3)
A_84_P863449	9.62	AT2G47770	TspO-like stress sensory protein (AtTSPO)
A_84_P12315	9.46	AT1G64660	Putative cystathionine gamma-synthase
A_84_P20442	9.12	AT4G20800	Putative reticuline dehydrogenase
A_84_P767554	9.07	AT5G59330	Protein of unknown function
A_84_P19684	0.021	AT5G38960	Putative germin-like protein
A_84_P20216	0.054	AT3G05950	Putative germin-like protein
A_84_P231139	0.056	AT2G42060	Protein of unknown function. contains C1-type domain
A_84_P12863	0.058	AT4G13420	Putative potassium cation transporter (AtHAK5)
A_84_P765727	0.061	AT4G19512	Protein of unknown function
A_84_P295014	0.063	AT5G57625	Putative PR-1-like extracellular protein of unknown function
A_84_P23783	0.065	AT1G52820	Putative 2OG-Fe(II) oxygenase
A_84_P11921	0.080	AT4G18010	Inositol polyphosphate 5-phosphatase (At5PTase2)
A_84_P22161	0.081	AT3G24300	Ammonium transporter 1;3 (AtAMT1;3)
A_84_P116942	0.082	AT4G02270	Putative (Ole e 1)-allergen-type protein of unknown function
A_84_P298044	0.083	AT2G28270	Protein of unknown function. contains C1-type domain
A_84_P167953	0.087	AT4G33880	Putative bHLH-type transcription factor
A_84_P127531	0.090	AT1G21310	Putative extensin-type glycoprotein (AtEXT3/AtEXT5)
A_84_P14045	0.091	AT5G48430	Putative aspartyl protease
A_84_P12305	0.099	AT1G52050	Putative jacalin-type lectin
A_84_P861057	0.102	AT1G12110	Dual-affinity nitrate transporter (AtNRT1.1/AtCHL1)
A_84_P19532	0.107	AT4G28850	Xyloglucan endotransglucosylase-hydrolase 26 (AtXTH26/AtXTH18)
A_84_P15735	0.110	AT4G26010	Putative class-III peroxidase (AtPer44)
A_84_P11438	0.110	AT1G78000	Putative sulfate transporter (AtSultr1.2)
A_84_P96076	0.112	AT3G25790	Putative GARP-G2-type transcription factor

Additional file 3.

List of the 20 most up and down regulated genes from *H. incana* shoots after 3 days of Pb exposure (100 µM $Pb(NO_3)_2$ in hydroponic cultures). Fold change control versus treated was validated by a Student test ($p<0.01$).

ProbeName	FC	AGI	Description
A_84_P767554	66.37	AT5G59330	Protein of unknown function
A_84_P863449	27.01	AT2G47770	TspO-like stress sensory protein (AtTSPO)
A_84_P784679	25.84	AT5G45690	Protein of unknown function
A_84_P12638	25.32	AT3G08860	Putative alanine-glyoxylate aminotransferase (AtPYD4)
A_84_P22091	23.87	AT3G03470	Cytochrome P450 monooxygenase (AtCYP89A9)
A_84_P580504	17.99	AT4G30050	Protein of unknown function
A_84_P849870	17.64	AT4G33110	Putative cyclopropane fatty acid synthase
A_84_P19758	17.31	AT5G59310	Lipid transfer protein (AtLTP4)
A_84_P14899	17.09	AT5G10930	Putative SNF1-related protein kinase (AtSnRK3.24/AtPKS19/AtCIPK5)
A_84_P825792	16.66	AT4G17030	Putative expansin (AtEXPR1)
A_84_P16611	16.27	AT4G02280	Sucrose synthase/sucrose-UDP glucosyltransferase (AtSUS3)
A_84_P798436	16.26	AT1G80130	Protein of unknown function
A_84_P13022	14.13	AT5G15250	ATP-dependent metalloprotease (AtFtsH6)
A_84_P12931	13.77	AT4G33150	Bifunctional lysine-ketoglutarate reductase & saccharopine dehydrogenase (AtLKR-SDH)
A_84_P21802	12.01	AT1G78780	Protein of unknown function
A_84_P605408	11.22	AT4G12580	Protein of unknown function
A_84_P220938	11.12	AT1G02470	Putative polyketide cyclase/dehydrase
A_84_P12620	9.93	AT2G02990	Ribonuclease T2 (AtRNS1)
A_84_P21822	9.47	AT1G21000	Putative PLATZ-type transcription factor
A_84_P197194	9.13	AT4G22870	Anthocyanidin synthase (AtANS)
A_84_P785414	0.103	AT4G16980	Putative classical arabinogalactan protein (AtAGP58C)
A_84_P20950	0.129	AT1G72610	Putative germin-like protein (AtGER1/AtGLP1)
A_84_P12763	0.134	AT3G50560	Putative NAD- or NADP-dependent oxidoreductase
A_84_P19997	0.136	AT1G74670	Putative GASA/GAST/Snakin-type gibberellin-regulated protein
A_84_P809616	0.145	AT2G45180	Putative seed storage/lipid transfer protein
A_84_P854454	0.147	AT2G10940	Putative proline-rich glycoprotein (AtPRP15)
A_84_P10193	0.157	AT5G18600	Putative glutaredoxin
A_84_P809513	0.159	AT5G14740	Beta carbonic anhydrase 2 (AtCA2/AtBCA2/AtCA18)
A_84_P13804	0.172	AT4G12420	Putative multi-copper oxidase (AtSKU5)
A_84_P21458	0.173	AT4G37610	BTB and TAZ domain protein 5 (AtBTB5)
A_84_P841591	0.182	AT3G23530	Putative cyclopropane fatty acid synthase
A_84_P14853	0.194	AT4G15480	UDP-dependent glycosyl transferase (AtUGT84A1)
A_84_P16770	0.196	AT5G04970	Putative pectinesterase (AtPME47)
A_84_P809692	0.200	AT3G16240	Putative tonoplast intrinsic protein (AtTIP2.1)
A_84_P16974	0.208	AT5G20630	Putative germin-like protein (AtGER3/AtGLP3)
A_84_P11031	0.208	AT4G30610	Putative serine carboxypeptidase (AtSCPL24/AtBRS1)
A_84_P12029	0.211	AT4G37220	Putative cold-responsive protein
A_84_P21069	0.213	AT2G06850	Xyloglucan endotransglucosylase-hydrolase (AtXTH4)
A_84_P10906	0.214	AT3G58120	Putative bZIP-type transcription factor (AtbZIP61)
A_84_P15764	0.215	AT4G32460	Protein of unknown function

Additional file 4.

List of the 20 most up- and down-regulated genes from *A. thaliana* roots after 3 days of Pb exposure (40 µM $Pb(NO_3)_2$ in hydroponic culture). The fold change control versus treated was validated by a Student test (P<0.01).

Probe Name	FC	AGI	Description
A_84_P18477	233.41	AT3G60120	Putative beta-glycosyl hydrolase (AtBGLU27)
A_84_P225679	199.37	AT5G39120	Putative germin-like protein
A_84_P21551	149.69	AT5G24540	Putative beta-glycosyl hydrolase (AtBGLU31)
A_84_P12477	144.51	AT1G79680	Putative cell wall-associated receptor-like protein kinase (AtWAKL10)
A_84_P23768	129.22	AT1G30100	Putative ABA biosynthesis carotenoid cleavage dioxygenase (AtNCED5)
A_84_P752326	124.87	AT1G62420	Protein of unknown function
A_84_P551677	123.39	AT2G02320	Putative PhloemProtein2-type protein of unknown function (AtPP2-B7)
A_84_P766178	119.68	AT5G39180	Putative germin-like protein
A_84_P513580	103.34	AT2G16005	Protein of unknown function. contains ML lipid recognition domain
A_84_P812392	93.65	AT4G33710	Putative PR-1-like extracellular protein of unknown function
A_84_P523886	91.47	AT5G46960	Putative invertase/pectin methylesterase inhibitor
A_84_P511454	84.83	AT5G43570	Putative PR-6 proteinase inhibitor
A_84_P214688	82.80	AT4G11340	Protein of unknown function
A_84_P14441	80.81	AT2G29460	Putative class tau glutathione S-transferase (AtGSTU4)
A_84_P558829	80.44	AT2G29350	Putative tropinone dehydrogenase-type oxidoreductase (AtSAG13)
A_84_P12563	80.27	AT2G44460	Putative beta-glycosyl hydrolase (AtBGLU28)
A_84_P10439	76.32	AT1G09080	Putative molecular chaperon (AtBiP-3/AtBP3)
A_84_P21883	68.86	AT1G68320	Putative Myb-type transcription factor (AtMYB62)
A_84_P140609	68.69	AT4G31970	Cytochrome P450 monooxygenase (AtCYP82C2)
A_84_P15416	68.37	AT2G36970	UDP-dependent glycosyl transferase (AtUGT86A1)
A_84_P21599	0.016	AT5G45230	Putative TIR-NBS-LRR class disease resistance protein
A_84_P11928	0.017	AT4G19800	Putative chitinase-type glycosyl hydrolase
A_84_P16526	0.017	AT3G46410	Protein of unknown function
A_84_P61030	0.018	AT5G28615	Protein of unknown function
A_84_P519134	0.018	AT3G50450	Hypersensitive response protein (AtHR1)
A_84_P11341	0.019	AT1G33820	Protein of unknown function
A_84_P18740	0.022	AT5G39620	Putative RAB-G-class small GTPase (AtRAB-G1)
A_84_P840330	0.024	AT1G50050	Putative PR-1-like extracellular protein of unknown function
A_84_P517200	0.025	AT2G31310	putative ASL/LBD-type transcription factor (AtASL17/AtLBD14)
A_84_P14663	0.025	AT3G52970	CYP76G1; electron carrier/ heme binding / monooxygenase/ oxygen binding
A_84_P513598	0.029	AT2G22122	Protein of unknown function
A_84_P11461	0.029	AT1G33930	Putative immune-associated GTP-binding protein (AtIAN6)
A_84_P237653	0.030	AT2G37800	Protein of unknown function. contains C1-type domain
A_84_P841699	0.031	AT1G33840	Protein of unknown function
A_84_P16681	0.031	AT4G26050	Protein of unknown function. contains LRR domain (AtPIRL8)
A_84_P17407	0.031	AT3G19430	Putative extensin-type glycoprotein (AtEXT51)
A_84_P788157	0.032	AT2G34315	Protein of unknown function
A_84_P13415	0.032	AT1G79130	Putative auxin-responsive protein
A_84_P96556	0.033	AT1G19900	Putative glyoxal oxidase
A_84_P269770	0.033	AT2G17590	Protein of unknown function. contains C1-type domain

Additional file 5.

List of the 20 most up- and down-regulated genes from *A. thaliana* shoots after 3 days of Pb exposure (40 µM $Pb(NO_3)_2$ in hydroponic cultures). The fold change control versus treated was validated by a Student test (P<0.01).

Probe Name	FC	AGI	Description
A_84_P710346	9.71	AT2G47015	MIR408; miRNA
A_84_P12207	8.87	AT5G62040	PEBP-type growth and differentiation regulator (AtBFT)
A_84_P22136	6.94	AT3G16360	Histidine phosphotransfer protein involved in cytokinin signaling (AtAHP4)
A_84_P63250	6.78	AT3G53400	Putative methyltransferase
A_84_P55560	5.70	AT5G03190	Putative methyltransferase
A_84_P265830	4.34	AT4G14020	Putative RALF-type endogenous peptide
A_84_P12563	4.06	AT2G44460	Putative beta-glycosyl hydrolase (AtBGLU28)
A_84_P92139	3.68	AT4G37700	Protein of unknown function
A_84_P205068	3.33	AT1G17744	Protein of unknown function
A_84_P13885	3.24	AT4G35190	Protein of unknown function. involved in cytokinin activation pathway (AtLOG5)
A_84_P17125	3.10	AT1G10970	Putative divalent transition metal cation transporter (AtZIP4)
A_84_P13908	3.09	AT4G15460	Protein of unknown function
A_84_P11710	3.03	AT1G53080	Putative legume lectin-like protein
A_84_P819800	3.02	AT2G25625	Protein of unknown function
A_84_P604601	2.89	AT4G37140	Methyl esterase (AtMES20)
A_84_P280890	2.89	AT3G10320	Protein of unknown function
A_84_P18376	2.86	AT3G23730	Xyloglucan endotransglucosylase-hydrolase (AtXTH16)
A_84_P16901	2.86	AT5G54060	UDP-dependent glycosyl transferase (AtUGT79B1)
A_84_P764077	2.82	AT4G01060	Myb-type transcription factor. involved in epidermal cell differentiation (AtCPL3/AtETC3)
A_84_P12393	2.81	AT1G18860	Putative WRKY-type transcription factor (AtWRKY61)
A_84_P61030	0.027	AT5G28615	Protein of unknown function
A_84_P761076	0.056	AT3G55646	Protein of unknown function
A_84_P14144	0.071	AT1G28170	Putative sulphotransferase (AtSOT7)
A_84_P12471	0.116	AT1G11460	Putative membrane protein of unknown function
A_84_P603601	0.120	AT4G02850	Putative PhzC/PhzF-like phenazine biosynthesis
A_84_P761311	0.127	AT3G13403	Putative defensin-like protein
A_84_P255930	0.181	AT4G08210	Protein of unknown function. contains pentatricopeptide (PPR) repeat
A_84_P761404	0.191	AT3G21460	Putative glutaredoxin
A_84_P19255	0.207	AT1G01190	Cytochrome P450 monooxygenase involved in reproductive development (AtCYP78A8)
A_84_P721943	0.216	AT1G10460	Putative germin-like protein (AtGLP7)
A_84_P18890	0.227	AT2G16485	Protein of unknown function. contains GYF and SWIB/MDM2 domain
A_84_P23716	0.246	AT1G12980	AP2-type transcription factor (AtDRN/AtESR1)
A_84_P11893	0.256	AT4G04840	Methionine sulfoxide reductase B6 (AtMSRB6)
A_84_P93089	0.279	AT1G13650	Protein of unknown function
A_84_P12872	0.295	AT1G29270	Protein of unknown function
A_84_P607864	0.309	AT3G02885	Putative GASA/GAST/Snakin-type gibberellin-regulated protein (AtGASA5)
A_84_P15038	0.311	AT5G61350	Putative CrRLK1L-type receptor protein kinase
A_84_P526212	0.319	AT3G46880	Protein of unknown function
A_84_P20381	0.323	AT3G63110	Adenylate isopentenyltransferase/cytokinin synthase (AtIPT3)
A_84_P576326	0.325	AT5G53210	Putative bHLH-type transcription factor. SPEECHLESS (AtSPCH)

Additional file 6.

H. incana microarray validation. *H. incana* gene expression FC measured with microarray and qRT-PCR methods were compared. Gene expression level measured in roots (R) and shoots (S) of *H. incana* plantlets treated with 100µM $Pb(NO_3)_2$ compared to non-treated plantlets.

AGI	Description	Symbol	Organ	Microarray FC	qRT-PCR FC
At1g06650	2-oxoglutarate-dependent dioxygenase		R	4.81	2.24
At1g15180	MATE-related efflux carrier	DTX13	R	4.96	27.09
At1g22990	Heavy-metal-associated domain-containing protein	HIPP22	R	5.76	5.10
At2g26020	Plant defensin 1.2b	PDF1.2b	R	39.04	287.12
At2g28660	Copper-binding family protein		R	6.16	11.65
At3g12750	Divalent transition metal cation transporter	ZIP1	R	2.96	4.77
At4g08570	heavy-metal-associated domain-containing protein		R	13.92	15.76
At4g13420	Potassium cation transporter	HAK5	R	0.06	0.07
At4g17030	Putative expansin	EXLB1	R	25.23	54.13
At4g25100	Iron superoxide dismutase	FSD1	R	3.25	5.34
At4g35090	Catalase	CAT2	R	0.25	0.35
At5g01600	Ferretin non-haem iron storage protein	ATFER1	R	3.68	3.17
At5g02380	Type 2 metallothionein	MT2B	R	2.15	2.25
At5g06530	Subfamily G ABC-type transporter	WBC23	R	2.65	2.04
At5g09930	Subfamily F ABC-type transporter	GCN2	R	2.95	7.71
At5g11930	Putative glutaredoxin		R	3.64	3.77
At5g13580	Subfamily G ABC-type transporter	WBC6	R	4.18	1.97
At5g15410	Cyclic nucleotide and calmodulin regulated ion channel	DND1	R	2.27	1.47
At5g38960	Germin-like protein		R	0.02	0.01
At1g74670	Gibberellin-responsive protein		S	0.14	0.13
At1g77510	Protein disulfide isomerase-like	PDIL1-2	S	3.49	8.46
At2g36380	Subfamily G ABC-type transporter	PDR6	S	2.03	4.34
At2g45180	Putative seed storage/lipid transfer protein		S	0.15	0.24
At3g09390	Type 2 metallothionein	MT2A	S	4.58	8.28
At3g50560	Putative NAD- or NADP-dependant oxidoreductase		S	0.13	0.25
At3g59140	Subfamily C ABC-type transporter	MRP14	S	2.10	1.52
At4g11600	Glutathione peroxidase	GPX6	S	2.12	4.14
At4g33020	Divalent transition metal cation transporter	ZIP9	S	5.86	4.00
At5g01600	Ferretin non-haem iron storage protein	FER1	S	2.51	5.11
At5g09930	Subfamily F ABC-type transporter	GCN2	S	4.69	9.29
At5g18600	glutaredoxin family protein		S	0.16	0.43
At5g45690	Protein of unknown function		S	25.84	47.39
At5g59330	Protein of unknown function		S	66.37	87.19

Additional file 7.

A. thaliana microarray validation. *A. thaliana* gene expression FC measured with microarray and QPCR methods were compared. Gene expression level was measured in roots (R) and shoots (S) of *A. thaliana* plantlets treated with 40µM $Pb(NO_3)_2$ compared to non-treated plantlets.

AGI	Description	Symbol	Organ	Microarray FC	qRT-PCR FC
At2g28660	Protein of unknown function		R	1.75	2.81
At3g127500	Divalent transition metal transporter	ZIP1	R	6.37	17.09
At4g35090	Catalase	CAT2	R	0.50	0.95
At5g01600	Ferritin non-haem iron storage protein	FER1	R	1.93	4.25
At5g02380	Type 2 metallothionein	MT2B	R	2.81	5.01
At3g12750	Type 2 metallothionein	MT2A	S	1.86	1.20
At4g08570	Ferritin non-haem iron storage protein	FER1	S	1.18	1.07

Additional file 8.

List of genes regulated by Pb in *H. incana* roots relative to *A. thaliana* roots. Genes regulated for both species (*A. thaliana* and *H. incana*) are in bold.

AGI	FC	Description
AT2G26010	50.67	PDF1.3 (plant defensin 1.3)
AT2G26020	39.04	PDF1.2b (plant defensin 1.2b)
**AT4G17030**	**25.23**	**ATEXLB1 (ARABIDOPSIS THALIANA EXPANSIN-LIKE B1)**
AT3G02480	23.99	Late embryogenesis abundant protein (LEA)
**AT3G09950**	**20.02**	**Unknown protein**
AT5G66400	19.42	RAB18 (RESPONSIVE TO ABA 18)
AT1G52400	18.05	BGLU18 (BETA GLUCOSIDASE 18); catalytic/ cation binding / hydrolase. hydrolyzing O-glycosyl compounds
AT2G14610	16.63	PR1 (PATHOGENESIS-RELATED GENE 1)
AT5G07330	16.55	Unknown protein
**AT4G12580**	**14.18**	**Unknown protein**
AT4G08570	13.92	Heavy metal transport/detoxification superfamily protein
**AT5G10930**	**13.23**	**CIPK5 (CBL-INTERACTING PROTEIN KINASE 5); ATP binding / kinase/ protein kinase**
AT3G21370	11.34	Beta glucosidase 19 (BGLU19)
**AT4G02280**	**10.37**	**SUS3 (sucrose synthase 3); UDP-glycosyltransferase/ sucrose synthase/ transferase. transferring glycosyl groups**
**AT2G47770**	**9.62**	**Membrane-bound protein (AtTSPO)**
**AT1G64660**	**9.46**	**ATMGL (ARABIDOPSIS THALIANA METHIONINE GAMMA-LYASE); catalytic/ methionine gamma-lyase**
AT4G20800	9.12	FAD-binding domain-containing protein
AT4G25700	8.54	BETA-OHASE 1 (BETA-HYDROXYLASE 1); carotene beta-ring hydroxylase
**AT3G17110**	**8.11**	**Pseudogene. glycine-rich protein**
**AT4G12290**	**7.49**	**Copper amine oxidase family protein amine oxidase/ copper ion binding / quinone binding**
AT5G54270	7.42	LHCB3 (LIGHT-HARVESTING CHLOROPHYLL B-BINDING PROTEIN 3); structural molecule
AT5G47560	7.21	TDT (TONOPLAST DICARBOXYLATE TRANSPORTER); malate transmembrane transporter
AT1G29910	7.10	CAB3 (CHLOROPHYLL A/B BINDING PROTEIN 3); chlorophyll binding
AT1G32350	6.82	AOX1D (alternative oxidase 1D); alternative oxidase
AT1G29930	6.55	CAB1 (CHLOROPHYLL A/B BINDING PROTEIN 1); chlorophyll binding
AT3G53160	6.43	UGT73C7 (UDP-glucosyl transferase 73C7); UDP-glycosyltransferase/ transferase. transferring glycosyl groups
AT1G32770	6.33	ANAC012 (ARABIDOPSIS NAC DOMAIN CONTAINING PROTEIN 12); specific transcriptional repressor
AT2G28660	6.16	Copper-binding family protein
AT1G56600	6.09	AtGolS2 (Arabidopsis thaliana galactinol synthase 2); transferase. transferring glycosyl groups
**AT1G33030**	**5.94**	**O-methyltransferase family 2 protein**
AT3G57240	5.92	BG3 (BETA-1.3-GLUCANASE 3); cellulase/ hydrolase. hydrolyzing O-glycosyl compounds
**AT3G03170**	**5.90**	**Unknown protein**
AT1G22990	5.76	Heavy metal transport/detoxification superfamily protein
AT4G15530	5.64	PPDK (pyruvate orthophosphate dikinase); kinase/ pyruvate. phosphate dikinase
AT2G24210	5.61	TPS10 (terpene synthase 10); (E)-beta-ocimene synthase/ myrcene synthase
AT2G21970	5.54	SEP2 (STRESS ENHANCED PROTEIN 2); chlorophyll binding
AT3G54890	5.51	LHCA1; chlorophyll binding
**AT4G33150**	**5.47**	**lysine-ketoglutarate reductase/saccharopine dehydrogenase bifunctional enzyme**
AT5G28450	5.44	Chlorophyll A-B binding family protein
AT2G15780	5.36	Cupredoxin superfamily protein; electron carrier activity. copper ion binding;
AT2G34430	5.22	LHB1B1; chlorophyll binding
AT1G30100	5.21	NCED5 (NINE-CIS-EPOXYCAROTENOID DIOXYGENASE 5); 9-cis-epoxycarotenoid dioxygenase
**AT1G45249**	**5.19**	**ABF2 (ABSCISIC ACID RESPONSIVE ELEMENTS-BINDING FACTOR 2)**
AT2G34420	5.14	LHB1B2; chlorophyll binding
AT5G36970	5.14	NHL25 (NDR1/HIN1-LIKE 25)
**AT2G02990**	**5.13**	**RNS1 (RIBONUCLEASE 1); endoribonuclease/ ribonuclease**
AT3G47470	5.02	LHCA4 (LIGHT-HARVESTING CHLOROPHYLL-PROTEIN COMPLEX I SUBUNIT A4); chlorophyll binding
AT1G15180	4.96	MATE efflux family protein
**AT1G53580**	**4.95**	**GLY3 (GLYOXALASE II 3); hydrolase/ hydroxyacylglutathione hydrolase**
AT1G06570	4.93	Phytoene desaturation 1
AT4G37970	4.84	CAD6 (CINNAMYL ALCOHOL DEHYDROGENASE 6); binding / catalytic/ oxidoreductase/ zinc ion binding
AT1G06650	4.81	2-oxoglutarate-dependent dioxygenase. putative
**AT4G19760**	**4.80**	**Catalytic/ cation binding / chitinase/ hydrolase. hydrolyzing O-glycosyl compounds**
AT3G47340	4.70	ASN1 (GLUTAMINE-DEPENDENT ASPARAGINE SYNTHASE 1)
AT1G26390	4.60	FAD-binding domain-containing protein
AT1G52342	4.59	Unknown protein
AT5G06860	4.58	ATPGIP1 (POLYGALACTURONASE INHIBITING PROTEIN 1)
**AT4G11650**	**4.45**	**ATOSM34 (osmotin 34)**
**AT5G57050**	**4.38**	**ABI2 (ABA INSENSITIVE 2); protein serine/threonine phosphatase**
AT1G51400	4.38	Photosystem II 5 kD protein
AT4G22530	4.38	Embryo-abundant protein-related
**AT2G44130**	**4.27**	**Galactose oxidase/kelch repeat superfamily protein**
AT1G58270	4.23	ZW9
AT1G08080	4.23	ACA7 (ALPHA CARBONIC ANHYDRASE 7); carbonate dehydratase/ zinc ion binding
**AT2G30570**	**4.20**	**PSBW (PHOTOSYSTEM II REACTION CENTER W)**
AT2G46770	4.19	EMB2301 (EMBRYO DEFECTIVE 2301); transcription activator/ transcription factor
**AT5G13580**	**4.18**	**ABC-2 type transporter family protein**
AT1G60420	4.00	DC1 domain-containing protein
AT1G21680	3.96	DPP6 N-terminal domain-like protein

AT4G19810	3.95	Glycosyl hydrolase family 18 protein
**AT4G26080**	**3.90**	**ABI1 (ABA INSENSITIVE 1); calcium ion binding**
**AT1G72770**	**3.89**	**HAB1 (HOMOLOGY TO ABI1); catalytic/ protein serine/threonine phosphatase**
**AT3G13672**	**3.81**	**TRAF-like superfamily protein**
AT4G28370	3.80	Protein binding / zinc ion binding
AT4G37370	3.77	CYP81D8; electron carrier/ heme binding / iron ion binding / monooxygenase/ oxygen binding
**AT5G16030**	**3.76**	**Unknown protein**
AT5G65230	3.64	AtMYB53 (myb domain protein 53); DNA binding / transcription factor
**AT4G24910**	**3.59**	**Unknown protein**
AT2G43580	3.54	Chitinase family protein
AT1G68320	3.50	MYB62 (myb domain protein 62); DNA binding / transcription factor
**AT3G11410**	**3.45**	**PP2CA (ARABIDOPSIS THALIANA PROTEIN PHOSPHATASE 2CA); protein binding / protein serine**
AT4G38410	3.45	Dehydrin family protein
AT3G28520	3.45	AAA-type ATPase family protein
AT3G03660	3.44	WOX11 (WUSCHEL related homeobox 11); DNA binding / transcription factor
**AT1G17870**	**3.43**	**EGY3 (ETHYLENE-DEPENDENT GRAVITROPISM-DEFICIENT AND YELLOW-GREEN-LIKE 3)**
AT4G25300	3.43	Oxidoreductase. 2OG-Fe(II) oxygenase family protein
AT2G31560	3.42	Unknown protein
AT1G22710	3.38	SUC2 (SUCROSE-PROTON SYMPORTER 2); carbohydrate transmembrane transporter
AT1G60970	3.38	Clathrin adaptor complex small chain family protein
AT5G37478	3.36	TPX2 (targeting protein for Xklp2) protein family
AT2G04038	3.35	AtbZIP48 (Arabidopsis thaliana basic leucine-zipper 48)
AT1G55910	3.33	ZIP11 (ZINC TRANSPORTER 11 PRECURSOR); metal ion transmembrane transporter
AT2G36800	3.33	DOGT1 (DON-GLUCOSYLTRANSFERASE 1)
**AT1G51140**	**3.31**	**basic helix-loop-helix (bHLH) family protein**
AT1G09610	3.31	Unknown protein
AT3G48770	3.24	ATP binding / DNA binding
AT3G26150	3.24	Putative cytochrome P450
AT3G24310	3.20	MYB305 (myb domain protein 305); DNA binding / transcription factor
AT3G26200	3.16	CYP71B22; electron carrier/ heme binding / iron ion binding / monooxygenase/ oxygen binding
AT5G03990	3.16	Unknown protein
AT3G57950	3.15	Unknown protein
AT5G24090	3.14	Chitinase A (class III) expressed exclusively under environmental stress conditions
**AT5G47720**	**3.14**	**acetyl-CoA C-acyltransferase. putative / 3-ketoacyl-CoA thiolase. putative**
AT1G62380	3.14	ACO2 (ACC OXIDASE 2); 1-aminocyclopropane-1-carboxylate oxidase
AT4G37360	3.13	CYP81D2; electron carrier/ heme binding / iron ion binding / monooxygenase/ oxygen binding
AT4G23250	3.13	EMBRYO DEFECTIVE 1290 (EMB1290); kinase/ protein kinase
AT4G04880	3.12	Adenosine/AMP deaminase family protein
AT3G60140	3.11	Glycosyl hydrolase superfamily protein
AT2G03590	3.09	ATUPS1 (ARABIDOPSIS THALIANA UREIDE PERMEASE 1); allantoin uptake transmembrane transporter
AT5G47810	3.03	PFK2 (PHOSPHOFRUCTOKINASE 2); 6-phosphofructokinase
AT1G63910	3.03	AtMYB103 (myb domain protein 103); DNA binding / transcription activator/ transcription factor
AT1G08630	3.01	THA1 (Threonine Aldolase 1); aldehyde-lyase/ threonine aldolase
AT1G75730	2.99	Unknown protein
AT3G12750	2.96	ZIP1 (ZINC TRANSPORTER 1 PRECURSOR); zinc ion transmembrane transporter
**AT5G09930**	**2.95**	**ATGCN2; transporter**
AT1G08290	2.94	Zinc finger (C2H2 type) protein (WIP3)
AT1G78070	2.93	Transducin/WD40 repeat-like superfamily protein
AT5G47640	2.93	NF-YB2 (NUCLEAR FACTOR Y. SUBUNIT B2); transcription factor
AT5G56870	2.86	BGAL4 (beta-galactosidase 4); beta-galactosidase
AT1G73830	2.86	BR enhanced expression 3 (BEE3); DNA binding / transcription factor
AT3G26190	2.86	CYP71B21; electron carrier/ heme binding / iron ion binding / monooxygenase/ oxygen binding
**AT3G61450**	**2.84**	**SYP73 (SYNTAXIN OF PLANTS 73); protein transporter**
AT1G19630	2.84	CYP722A1; electron carrier/ heme binding / iron ion binding / monooxygenase/ oxygen binding
AT3G05165	2.84	Sugar transporter. putative
AT2G22150	2.82	Pseudogene. hypothetical protein
AT1G12200	2.79	Flavin-binding monooxygenase family protein
AT5G17450	2.76	Heavy metal transport/detoxification superfamily protein
AT5G64552	2.72	CPuORF22 (Conserved peptide upstream open reading frame 22)
**AT3G22600**	**2.71**	**Bifunctional inhibitor/lipid-transfer protein/seed storage 2S albumin superfamily protein**
AT4G30975	2.71	Unknown gene
AT4G18350	2.69	NCED2 (NINE-CIS-EPOXYCAROTENOID DIOXYGENASE 2); 9-cis-epoxycarotenoid dioxygenase
AT5G45020	2.68	Glutathione S-transferase family protein
AT4G13670	2.67	PTAC5 (PLASTID TRANSCRIPTIONALLY ACTIVE5); heat shock protein binding / unfolded protein binding
AT3G15500	2.66	ANAC055 (ARABIDOPSIS NAC DOMAIN CONTAINING PROTEIN 55); transcription factor
AT4G34588	2.66	GBF6 (G-BOX BINDING FACTOR 6); DNA binding / protein heterodimerization/ transcription factor
AT5G06530	2.65	ABC-2 type transporter family protein
AT4G39700	2.62	Heavy metal transport/detoxification superfamily protein
AT4G34350	2.61	4-hydroxy-3-methylbut-2-en-1-yl diphosphate reductase
AT4G27435	2.60	Unknown protein
AT5G13370	2.59	Auxin-responsive GH3 family protein
AT1G56220	2.59	Dormancy/auxin associated family protein
AT3G21500	2.56	1-deoxy-D-xylulose-5-phosphate synthase
AT5G25890	2.55	IAA28 (INDOLE-3-ACETIC ACID INDUCIBLE 28); transcription factor
AT5G62530	2.55	Encodes mitochondrial Delta-pyrroline-5-carboxylate dehydrogenase
AT2G35060	2.53	KUP11; potassium ion transmembrane transporter
**AT3G59140**	**2.52**	**ATMRP14; ATPase. coupled to transmembrane movement of substances**

AT1G67710	2.52	ARR11 (RESPONSE REGULATOR 11); transcription factor/ two-component response regulator
AT4G23260	2.50	ATP binding / protein kinase/ protein serine/threonine kinase/ protein tyrosine kinase
**AT1G19650**	**2.50**	**SEC14 cytosolic factor. putative / phosphoglyceride transfer protein. putative**
AT5G54400	2.47	S-adenosyl-L-methionine-dependent methyltransferases superfamily protein
AT1G55760	2.45	BTB/POZ domain-containing protein
AT3G10450	2.44	SCPL7 (SERINE CARBOXYPEPTIDASE-LIKE 7); serine-type carboxypeptidase
AT4G39830	2.44	Cupredoxin superfamily protein
AT1G07380	2.43	Ceramidase family protein
AT4G04630	2.42	Unknown protein
AT5G17420	2.42	IRX3 (IRREGULAR XYLEM 3); cellulose synthase
AT3G50030	2.42	ARM-repeat/Tetratricopeptide repeat (TPR)-like protein
AT5G44720	2.40	Molybdenum cofactor sulfurase family protein
**AT4G26580**	**2.37**	**Protein binding / zinc ion binding**
AT5G55250	2.35	Indole acetic acid carboxylmethyltransferase (IAMT1)
AT2G16895	2.35	Pseudogene
AT5G24105	2.31	AGP41 (ARABINOGALACTAN-PROTEIN 41)
AT4G37890	2.28	EDA40 (embryo sac development arrest 40); protein binding / ubiquitin-protein ligase/ zinc ion binding
AT5G15410	2.27	DND1 (DEFENSE NO DEATH 1); calcium channel/ calmodulin binding / cation channel
AT1G75030	2.25	ATLP-3
AT1G10370	2.21	ERD9 (EARLY-RESPONSIVE TO DEHYDRATION 9); glutathione transferase
AT2G47240	2.21	Long-chain-fatty-acid--CoA ligase family protein / long-chain acyl-CoA synthetase family protein
AT3G27400	2.20	Pectate lyase family protein
AT3G59690	2.20	IQD13 (IQ-domain 13); calmodulin binding
AT3G04720	2.19	PR4 (PATHOGENESIS-RELATED 4); chitin binding
AT5G46240	2.17	KAT1 (POTASSIUM CHANNEL IN ARABIDOPSIS THALIANA 1); cyclic nucleotide binding
AT3G49120	2.16	Class III peroxidase Perx34
AT3G59050	2.16	ATPAO3 (Polyamine oxidase 3)
AT1G69920	2.16	ATGSTU12 (GLUTATHIONE S-TRANSFERASE TAU 12); glutathione transferase
AT5G18850	2.14	Unknown protein
AT1G67070	2.13	DIN9 (DARK INDUCIBLE 9); mannose-6-phosphate isomerase
AT5G55420	2.11	Encodes a Protease inhibitor/seed storage/LTP family protein [pseudogene]
AT1G23800	2.11	ALDH2B7; 3-chloroallyl aldehyde dehydrogenase/ aldehyde dehydrogenase (NAD)
AT4G17980	2.09	Anac071 (Arabidopsis NAC domain containing protein 71); transcription factor
AT4G23410	2.08	TET5 (TETRASPANIN5)
AT5G59780	2.07	MYB59 (MYB DOMAIN PROTEIN 59); DNA binding / transcription factor
AT5G39050	2.06	Transferase/ transferase. transferring acyl groups other than amino-acyl groups
AT1G52570	2.06	PLDALPHA2 (phosphlipase d alpha 2); phospholipase D
AT2G32800	2.05	AP4.3A; ATP binding / protein kinase/ protein serine/threonine kinase/ protein tyrosine kinase
AT5G06510	2.05	NF-YA10 (NUCLEAR FACTOR Y. SUBUNIT A10); transcription factor
AT5G54980	2.05	Unknown protein
AT2G37090	2.04	IRX9 (IRREGULAR XYLEM 9); transferase. transferring glycosyl groups / xylosyltransferase
AT5G18150	0.499	Methyltransferase-related protein
AT5G59080	0.498	Unknown protein
AT1G18870	0.495	ICS2 (ISOCHORISMATE SYNTHASE 2); isochorismate synthase
AT4G10640	0.494	IQD16 (IQ-domain 16); calmodulin binding
AT3G44735	0.492	Phytosulfokine 3 precursor. coding for a unique plant peptide growth factor
AT1G01280	0.491	CYP703A2 (CYTOCHROME P450. FAMILY 703. SUBFAMILY A. POLYPEPTIDE 2)
AT2G40270	0.485	Protein kinase family protein
AT4G02380	0.480	Encodes AtLEA5 (late embryogenesis abundant like protein)
AT2G39040	0.479	Peroxidase superfamily protein
AT1G25240	0.474	Epsin N-terminal homology (ENTH) domain-containing protein
AT2G44790	0.467	UCC2 (UCLACYANIN 2); copper ion binding / electron carrier
AT2G37260	0.466	TTG2 (TRANSPARENT TESTA GLABRA 2); transcription factor
AT2G40970	0.464	myb family transcription factor (MYBC1)
AT1G66970	0.463	SVL2 (SHV3-LIKE 2); glycerophosphodiester phosphodiesterase/ kinase
AT4G37520	0.463	Peroxidase superfamily protein
AT1G26240	0.458	Proline-rich extensin-like family protein
AT2G28950	0.456	ATEXPA6 (ARABIDOPSIS THALIANA EXPANSIN A6)
AT3G16400	0.453	NSP1 (NITRILE SPECIFIER PROTEIN 1)
AT1G06620	0.450	2-oxoglutarate-dependent dioxygenase. putative
AT5G28640	0.450	AN3 (ANGUSTIFOLIA 3); protein binding / transcription coactivator
AT1G77330	0.450	1-aminocyclopropane-1-carboxylate oxidase. putative / ACC oxidase. putative
AT1G57590	0.445	Pectinacetylesterase family protein
**AT3G16370**	**0.440**	**GDSL-motif lipase/hydrolase family protein**
AT5G16010	0.440	3-oxo-5-alpha-steroid 4-dehydrogenase family protein
AT1G18390	0.439	ATP binding / kinase/ protein kinase/ protein serine/threonine kinase
AT4G22485	0.439	Encodes a Protease inhibitor/seed storage/LTP family protein
AT5G40780	0.437	LHT1; amino acid transmembrane transporter
AT5G16530	0.435	PIN5 (PIN-FORMED 5); auxin:hydrogen symporter/ transporter
AT2G15500	0.428	Poly(A) binding protein 2
AT4G06521	0.427	Transposable element gene
AT4G11310	0.426	Cysteine proteinase. putative
**AT4G03210**	**0.423**	**XTH9 (Xyloglucan endotransglucosylase/hydrolase 9)**
AT1G62660	0.417	Beta-fructosidase (BFRUCT3) / beta-fructofuranosidase / invertase. vacuolar
AT3G15370	0.416	ATEXPA12 (ARABIDOPSIS THALIANA EXPANSIN 12)
AT1G10960	0.414	Ferredoxin 1 (FD1) ATFD1 (FERREDOXIN 1); 2 iron. 2 sulfur cluster binding
AT4G08410	0.406	Proline-rich extensin-like family protein

AT1G75250	0.405	ATRL6 (ARABIDOPSIS RAD-LIKE 6); transcription factor
AT4G21870	0.399	26.5 kDa class P-related heat shock protein (HSP26.5-P)
AT1G09560	0.398	Germin-like protein (GLP5) ; manganese ion binding / nutrient reservoir
AT4G04920	0.397	SFR6 (SENSITIVE TO FREEZING 6)
AT5G53250	0.395	AGP22 (ARABINOGALACTAN PROTEIN 22)
AT4G08850	0.392	Leucine-rich repeat receptor-like protein kinase family protein
AT5G24270	0.391	SOS3 (SALT OVERLY SENSITIVE 3); calcium ion binding / calcium-dependent protein serine
AT4G27260	0.389	WES1; indole-3-acetic acid amido synthetase
AT3G09810	0.389	Isocitrate dehydrogenase. putative / NAD+ isocitrate dehydrogenase. putative
AT3G47110	0.387	Leucine-rich repeat transmembrane protein kinase. putative
AT2G28650	0.383	ATEXO70H8 (exocyst subunit EXO70 family protein H8)
AT5G64410	0.375	OPT4 (OLIGOPEPTIDE TRANSPORTER 4); oligopeptide transporter
AT1G59590	0.374	ZCF37
AT5G19100	0.365	Extracellular dermal glycoprotein-related / EDGP-related
AT3G54040	0.350	Photoassimilate-responsive protein-related
AT5G08240	0.348	Unknown protein
AT5G06200	0.347	Unknown protein
AT5G44460	0.345	Calmodulin like 43 (CML43)
AT3G55110	0.341	ABC-2 type transporter family protein
AT1G01200	0.339	ATRABA3 (ARABIDOPSIS RAB GTPASE HOMOLOG A3); GTP binding
**AT5G44020**	**0.336**	**Acid phosphatase class B family protein**
AT3G47980	0.332	Integral membrane HPP family protein
AT2G39380	0.331	ATEXO70H2 (EXOCYST SUBUNIT EXO70 FAMILY PROTEIN H2)
AT1G61475	0.327	ATP binding / protein kinase
AT5G20110	0.327	Dynein light chain type 1 family protein
AT3G15700	0.323	P-loop containing nucleoside triphosphate hydrolases superfamily protein
AT3G28550	0.321	Proline-rich extensin-like family protein
AT3G51680	0.318	Short-chain dehydrogenase/reductase (SDR) family protein
AT3G06070	0.318	Unknown protein
AT4G05390	0.315	ATRFNR1 (ROOT FNR 1); FAD binding / NADP or NADPH binding / electron carrier
AT1G21130	0.315	O-methyltransferase family protein
AT4G23980	0.313	ARF9 (AUXIN RESPONSE FACTOR 9); transcription factor
AT1G74440	0.311	Unknown protein
AT1G52810	0.311	2-oxoglutarate-dependent dioxygenase-related
AT5G45280	0.310	Pectinacetylesterase family protein
AT4G25790	0.310	Allergen V5/Tpx-1-related family protein
AT5G57010	0.309	Calmodulin-binding family protein
AT4G27730	0.308	OPT6 (OLIGOPEPTIDE TRANSPORTER 1); oligopeptide transporter
AT4G31250	0.301	Leucine-rich repeat transmembrane protein kinase. putative
AT3G20090	0.295	Member of CYP705A CYP705A18; electron carrier/ heme binding / iron ion binding
AT3G27170	0.292	CLC-B (Chloride channel B)
**AT1G70410**	**0.290**	**Carbonic anhydrase. putative / carbonate dehydratase. putative**
AT4G31470	0.289	Pathogenesis-related protein. putative
AT2G24980	0.280	Proline-rich extensin-like family protein
AT2G22750	0.278	Basic helix-loop-helix (bHLH) family protein
AT2G32660	0.275	AtRLP22 (Receptor Like Protein 22); kinase/ protein binding
AT5G49665	0.272	Zinc finger (C3HC4-type RING finger) family protein
AT1G21550	0.271	Calcium-binding protein. putative
AT3G17050	0.269	Transposable element gene
AT4G24310	0.265	Unknown protein
AT5G36920	0.263	Unknown protein
AT5G53160	0.262	Encodes RCAR3. a regulatory component of ABA receptor
AT5G65980	0.259	Auxin efflux carrier family protein
AT2G34180	0.256	CBL-interacting protein kinase 13 (CIPK13
AT2G16430	0.251	PAP10 (PURPLE ACID PHOSPHATASE 10); acid phosphatase/ protein serine
AT4G01220	0.250	Nucleotide-diphospho-sugar transferase family protein
AT3G13784	0.250	AtcwINV5 (Arabidopsis thaliana cell wall invertase 5)
AT2G40260	0.248	Myb family transcription factor
AT2G36325	0.247	GDSL-like Lipase/Acylhydrolase superfamily protein
AT4G37320	0.245	CYP81D5; electron carrier/ heme binding / iron ion binding / monooxygenase
AT3G61880	0.244	CYP78A9 (CYTOCHROME P450 78A9); monooxygenase/ oxygen binding
AT5G03545	0.237	Unknown protein
AT1G18140	0.235	LAC1 (Laccase 1); laccase
AT1G49030	0.229	PLAC8 family protein
AT3G47740	0.229	ATATH2; ATPase. coupled to transmembrane movement of substances
AT3G24503	0.223	ALDH2C4; 3-chloroallyl aldehyde dehydrogenase/ aldehyde dehydrogenase (NAD)
AT5G47740	0.222	Adenine nucleotide alpha hydrolases-like superfamily protein
AT1G30510	0.222	ATRFNR2 (ROOT FNR 2); FAD binding / NADP or NADPH binding / electron carrier
AT3G12700	0.221	Aspartyl protease family protein
AT1G24530	0.213	Transducin family protein / WD-40 repeat family protein
AT3G09220	0.210	LAC7 (laccase 7); laccase
AT3G25930	0.210	Adenine nucleotide alpha hydrolases-like superfamily protein
AT1G16530	0.210	ASL9 (ASYMMETRIC LEAVES 2 LIKE 9)
AT2G28670	0.207	Disease resistance-responsive (dirigent-like protein) family protein
AT2G15620	0.207	NIR1 (NITRITE REDUCTASE 1); ferredoxin-nitrate reductase/ nitrite reductase (NO-forming)
AT1G76990	0.203	ACR3; amino acid binding
AT1G14820	0.203	SEC14 cytosolic factor family protein / phosphoglyceride transfer family protein

AT4G30320	0.202	Allergen V5/Tpx-1-related family protein
AT1G30370	0.201	lipase class 3 family protein
AT5G60530	0.201	Late embryogenesis abundant protein-related / LEA protein-related
AT2G05520	0.198	GRP-3 (GLYCINE-RICH PROTEIN 3)
AT3G01420	0.196	DOX1; lipoxygenase
AT4G24730	0.193	Calcineurin-like phosphoesterase family protein
AT2G28780	0.189	Unknown protein
**AT3G52450**	**0.187**	**PUB22 (PLANT U-BOX 22); ubiquitin-protein ligase**
**AT4G37220**	**0.187**	**Cold acclimation protein WCOR413 family**
AT1G01750	0.182	ADF11 (ACTIN DEPOLYMERIZING FACTOR 11); actin binding
AT2G44370	0.180	Cysteine/Histidine-rich C1 domain family protein
AT1G66200	0.179	ATGSR2; copper ion binding / glutamate-ammonia ligase
AT3G25190	0.179	Vacuolar iron transporter (VIT) family protein
AT5G26310	0.175	UGT72E3; UDP-glycosyltransferase/ coniferyl-alcohol glucosyltransferase
AT3G62780	0.174	Calcium-dependent lipid-binding (CaLB domain) family protein
AT4G37160	0.168	Sks15 (SKU5 Similar 15); copper ion binding / oxidoreductase
AT2G37740	0.168	ZFP10 (ZINC-FINGER PROTEIN 10); nucleic acid binding / transcription factor/ zinc ion binding
AT1G51913	0.163	Unknown protein
AT3G16900	0.157	Unknown protein
AT2G23960	0.154	Defense-related protein. putative
AT5G50660	0.152	Unknown protein
AT2G23620	0.150	MES1 (METHYL ESTERASE 1); hydrolase. acting on ester bonds
AT5G14150	0.140	Unknown protein
AT5G37600	0.138	ATGSR1; copper ion binding / glutamate-ammonia ligase
AT2G34390	0.135	Aquaporin NIP2.1
AT2G43140	0.134	DNA binding / transcription factor
AT1G14160	0.134	Unknown protein
AT4G01630	0.131	ATEXPA17 (ARABIDOPSIS THALIANA EXPANSIN A17)
AT4G30280	0.130	XTH18 (XYLOGLUCAN ENDOTRANSGLUCOSYLASE/HYDROLASE 18)
AT5G14750	0.130	ATMYB66 (MYB DOMAIN PROTEIN 66); DNA binding / protein binding / transcription factor
AT1G70880	0.126	Bet v I allergen family protein
AT1G72416	0.125	Heat shock protein binding
**AT3G10720**	**0.120**	**Plant invertase/pectin methylesterase inhibitor superfamily**
AT1G73600	0.119	Methyltransferase/ phosphoethanolamine N-methyltransferase
**AT3G50560**	**0.118**	**Short-chain dehydrogenase/reductase (SDR) family protein**
AT3G25790	0.112	Myb family transcription factor
AT1G78000	0.110	SULTR1;2 (SULFATE TRANSPORTER 1;2); sulfate transmembrane transporter
AT1G12110	0.102	NRT1.1; nitrate transmembrane transporter/ transporter
AT1G52050	0.099	Mannose-binding lectin superfamily protein
AT1G21310	0.090	ATEXT3 (EXTENSIN 3); structural constituent of cell wall
AT2G28270	0.083	Cysteine/Histidine-rich C1 domain family protein
AT3G24300	0.081	AMT1;3 (AMMONIUM TRANSPORTER 1;3); ammonium transmembrane transporter
AT4G18010	0.080	AT5PTASE2 (MYO-INOSITOL POLYPHOSPHATE 5-PHOSPHATASE 2)
AT1G52820	0.065	2-oxoglutarate-dependent dioxygenase. putative
AT4G13420	0.058	HAK5 (HIGH AFFINITY K+ TRANSPORTER 5); potassium ion transmembrane transporter
AT2G42060	0.056	CHP-rich zinc finger protein. putative
AT3G05950	0.054	RmlC-like cupins superfamily protein; manganese ion binding. nutrient reservoir activity
AT5G38960	0.021	RmlC-like cupins superfamily protein; manganese ion binding. nutrient reservoir activity

Additional file 9.

List of genes regulated by Pb in *H. incana* shoots relative to *A. thaliana* shoots. Genes regulated for both species (*A. thaliana* and *H. incana*) are in bold.

AGI	FC	Description
AT4G16980	0.103	Arabinogalactan-protein family
AT1G72610	0.129	GER1 (Germin-like protein 1); oxalate oxidase
**AT3G50560**	**0.134**	**NAD(P)-binding Rossmann-fold superfamily protein**
AT1G74670	0.136	Gibberellin-responsive protein. putative
AT2G45180	0.145	Bifunctional inhibitor/lipid-transfer protein
AT2G10940	0.147	Bifunctional inhibitor/lipid-transfer protein
AT5G18600	0.157	Thioredoxin superfamily protein
AT3G01500	0.159	Beta carbonic anhydrase
AT5G14740	0.159	Beta carbonic anhydrase
AT4G12420	0.172	SKU5; copper ion binding / oxidoreductase
AT4G37610	0.173	BT5 (BTB AND TAZ DOMAIN PROTEIN 5); protein binding
AT3G23530	0.182	Cyclopropane fatty acid synthase. putative / CPA-FA synthase. putative
AT4G15480	0.194	UDP-dependent glycosyl transferase
AT5G04970	0.196	Plant invertase/pectin methylesterase inhibitor superfamily
AT3G16240	0.200	DELTA-TIP; ammonia transporter/ methylammonium transmembrane transporter
AT4G30610	0.208	BRS1 (BRI1 suppressor 1); serine-type carboxypeptidase
AT5G20630	0.208	GER3 (Germin 3); oxalate oxidase
**AT4G37220**	**0.211**	**Cold acclimation protein WCOR413 family**
AT2G06850	0.213	EXGT-A1 (Endoxyloglucan Transferase); hydrolase. acting on glycosyl bonds
AT3G58120	0.214	BZIP61; DNA binding / transcription activator
AT4G32460	0.215	Unknown protein
AT3G23730	0.224	Xyloglucan endotransglucosylase/hydrolase 16 (XTH16)
AT1G68590	0.230	Plastid-specific 30S ribosomal protein 3. putative / PSRP-3. putative
AT1G29070	0.235	Ribosomal protein L34 family protein
**AT3G52450**	**0.239**	**PUB22 (Plant U-BOX 22); ubiquitin-protein ligase**
**AT3G10720**	**0.240**	**Plant invertase/pectin methylesterase inhibitor superfamily**
AT2G03760	0.247	Sulphotransferase. putative
AT2G30010	0.248	TBL-type polysaccharide O-acetyltransferase. putative
AT2G36830	0.249	Tonoplast intrinsic protein. putative
AT2G05070	0.249	LHCB2.2; chlorophyll binding
AT5G45670	0.256	GDSL-motif lipase/hydrolase family protein
AT5G28770	0.261	BZO2H3; DNA binding / protein heterodimerization
AT4G18970	0.262	GDSL-motif lipase/hydrolase family protein
AT3G56650	0.266	Mog1/PsbP/DUF1795-like photosystem II reaction center PsbP family protein
AT1G23480	0.267	ATCSLA03 (Cellulose synthase-Like A3); cellulose synthase
AT5G25460	0.269	Unknown protein
AT4G22010	0.271	Type I multi-copper oxidase
AT3G03780	0.276	AtMS2; 5-methyltetrahydropteroyltriglutamate-homocysteine S-methyltransferase
**AT5G44020**	**0.277**	**Acid phosphatase class B family protein**
AT3G14770	0.282	Nodulin MtN3 family protein
AT1G59940	0.286	ARR3 (Response RegulatorR 3); transcription regulator
AT2G22330	0.290	CYP79B3; electron carrier/ heme binding / iron ion binding
AT3G26520	0.291	TIP2 (Tonoplast Intrinsic Protein 2); water channel
AT2G32690	0.292	GRP23 (Glycine-Rich Protein 23)
AT1G73110	0.299	P-loop containing nucleoside triphosphate hydrolases superfamily protein
**AT4G03210**	**0.302**	**XTH9 (Xyloglucan Endotransglucosidase /Hydrolase 9)**
AT1G03870	0.302	FLA9 (Fasciclin -Like Arabinogalactan 9)
AT2G18328	0.303	ATRL4 (Arabidopsis RAD-Like 4); DNA binding
AT3G04290	0.303	LTL1 (LI-TOLERANT LIPASE 1); carboxylesterase/ hydrolase. acting on ester bonds
**AT3G16370**	**0.304**	**GDSL-motif lipase/hydrolase family protein**
AT5G53490	0.309	Thylakoid lumenal 17.4 kDa protein. chloroplast
AT2G20562	0.311	Unknown protein
AT1G11850	0.316	Unknown protein
AT4G28780	0.318	GDSL-motif lipase/hydrolase family protein
AT5G45950	0.319	GDSL-motif lipase/hydrolase family protein
AT2G37220	0.320	29 kDa ribonucleoprotein. chloroplast. putative
AT1G78630	0.322	L13-type protein of large ribosomal subunit. putative
AT4G36540	0.326	BEE2 (BR Enhanced Expression 2); DNA binding
AT2G16500	0.331	ADC1 (Arginine Decarboxylase 1); arginine decarboxylase
AT1G04030	0.339	Unknown protein
**AT5G16030**	**0.346**	**Unknown protein**
AT5G50250	0.346	31 kDa ribonucleoprotein. chloroplast. putative / RNA-binding protein RNP-T. putative
AT4G20360	0.350	ATRABE1B (Arabidopsis RAB GTPase Homolog E1B)
AT1G72970	0.352	HTH (Hothead); FAD binding / aldehyde-lyase/ mandelonitrile lyase
AT1G14150	0.357	Oxygen evolving enhancer 3 (PsbQ) family protein
AT1G04680	0.357	Pectin lyase-like superfamily protein

AT1G56050	0.358	GTP-binding protein-related
AT5G25190	0.358	Ethylene-responsive element-binding protein. putative
AT2G28630	0.360	KCS12 (3-Ketoacyl -CoA Synthase 12); acyltransferase/ catalytic
AT1G70370	0.363	Polygalacturonase 2 (PG2)
AT4G19380	0.368	Long-chain fatty alcohol dehydrogenase family protein
AT4G29060	0.370	Plastidial Ts-type translation elongation factor. putative
AT1G10020	0.376	Unknown protein
AT3G15810	0.380	Unknown protein
AT3G54400	0.384	Aspartyl protease family protein
AT5G09650	0.384	AtPPa6 (Arabidopsis thaliana pyrophosphorylase 6)
AT1G71710	0.385	DNAse I-like superfamily protein
AT5G32616	0.387	Transposable element gene
AT4G25260	0.387	Invertase/pectin methylesterase inhibitor family protein
AT5G26670	0.388	Pectinacetylesterase. putative
AT1G69450	0.390	Early-responsive to dehydration stress protein (ERD4)
AT1G11860	0.393	Glycine cleavage T-protein family; aminomethyltransferase activity
AT4G00165	0.396	Bifunctional inhibitor/lipid-transfer protein
AT1G14810	0.396	Semialdehyde dehydrogenase family protein
AT3G27160	0.398	GHS1 (Glucose Hypersensitive 1); structural constituent of ribosome
AT5G47210	0.399	Nuclear RNA-binding protein. putative
AT3G19450	0.401	ATCAD4; cinnamyl-alcohol dehydrogenase
AT5G24850	0.403	CRY3 (cryptochrome 3); DNA binding / DNA photolyase/ FMN binding
AT3G10185	0.408	Gibberellin-regulated GASA/GAST/Snakin family protein
AT5G01240	0.409	LAX1 (Like Auxin Resistant)
AT5G03720	0.410	AT-HSFA3; DNA binding / transcription factor
AT2G24090	0.412	Ribosomal protein L35 family protein
AT1G48480	0.412	RKL1; ATP binding / kinase/ protein serine/threonine kinase
AT3G24450	0.412	Heavy metal transport/detoxification superfamily protein
AT3G12780	0.414	PGK1 (Phosphoglycerate Kinase 1); phosphoglycerate kinase
AT3G11630	0.416	2-cys peroxiredoxin. chloroplast (BAS1)
AT3G55330	0.418	PPL1 (PsbP-like protein 1); calcium ion binding
AT2G22230	0.421	Beta-hydroxyacyl-ACP dehydratase. putative
AT1G10522	0.423	Unknown protein
**AT1G70410**	**0.424**	**Beta-carbonic anhydrase betaCA4. putative**
AT1G56190	0.426	Phosphoglycerate kinase. putative
**AT2G30570**	**0.427**	**PSBW (Photosystem II Reaction Center W)**
AT4G34830	0.428	Regulator of ribulose-1.5-bisphosphate carboxylase/oxygenase
AT5G49030	0.429	OVA2 (ovule abortion 2); ATP binding / aminoacyl-tRNA ligase
AT1G54500	0.432	Rubredoxin family protein
AT1G65295	0.432	Unknown protein
AT2G07690	0.434	Minichromosome maintenance family protein / MCM family protein
AT3G28910	0.437	MYB30 (Myb Domain Protein 30); DNA binding / transcription factor
AT1G11870	0.437	SRS (Seryl -tRNA Synthetase); serine-tRNA ligase
AT3G59040	0.441	Pentatricopeptide (PPR) repeat-containing protein
AT3G27360	0.444	Histone H3
AT1G74330	0.444	ATP binding / protein kinase/ protein serine/threonine kinase
AT2G38140	0.445	PSRP4 (Plastid -Specific Ribosomal Protein 4); structural constituent of ribosome
AT2G47940	0.446	DEGP2; serine-type endopeptidase/ serine-type peptidase
AT4G24930	0.449	Thylakoid lumenal 17.9 kDa protein. chloroplast
AT1G73650	0.449	Oxidoreductase. acting on the CH-CH group of donors
AT2G35390	0.451	Ribose-phosphate pyrophosphokinase 1
AT5G12860	0.453	DiT1 (dicarboxylate transporter 1); oxoglutarate:malate antiporter
AT3G52500	0.453	Aspartyl protease family protein
AT3G50685	0.455	Unknown protein
AT3G17609	0.456	HYH (Hy5-Homolog); DNA binding / transcription factor
AT5G61170	0.456	40S ribosomal protein S19 (RPS19C)
AT2G16440	0.457	MCM-type helicase. involved in DNA replication. putative
AT3G60320	0.458	Unknown protein
AT1G07370	0.458	Proliferating cell nuclear antigen
AT1G66570	0.459	ATSUC7 (Sucrose-proton symporter 7)
AT3G48500	0.464	Component of plastidial RNA polymerase PEP complex. putative
AT5G52882	0.465	ATP binding / nucleoside-triphosphatase/ nucleotide binding
AT5G03300	0.466	ADK2 (Adenosine Kinase 2); adenosine kinase/ copper ion binding / kinase
AT5G14910	0.467	Heavy metal transport/detoxification superfamily protein
AT3G15520	0.470	Cyclophilin-like peptidyl-prolyl cis-trans isomerase family protein (SWEET2)
AT5G16190	0.471	ATCSLA11; cellulose synthase
AT3G63490	0.471	Ribosomal protein L1 family protein
AT3G46740	0.473	TOC75-III (Translocon at the outer envelope membrane of chloroplasts 75-III)
AT1G77490	0.475	TAPX (Thylakoidal Ascorbate Peroxydase); L-ascorbate peroxidase
AT5G44680	0.477	Methyladenine glycosylase family protein
AT5G21930	0.478	PAA2 (P-Type ATPase of Arabidopsis 2)
AT5G26820	0.480	ATIREG3 (Iron - Regulated Protein 3)
AT2G33430	0.484	DAL1 (Differentiation and Greening -Like 1)
AT5G23060	0.489	CaS (Calcium sensing receptor)

AT5G40160	0.491	EMB506 (embryo defective 506); protein binding
AT4G24810	0.494	ABC1 family protein
AT1G18250	0.496	Thaumatin-like protein (ATLP-1)
AT1G74910	0.498	ADP-glucose pyrophosphorylase family protein
AT3G54880	0.499	Unknown protein
AT5G23860	0.500	TUB8; structural constituent of cytoskeleton
ATMG00140	2.02	Unknown protein
AT1G61050	2.02	Alpha 1.4-glycosyltransferase family protein
AT3G61960	2.02	Protein kinase family protein
AT5G02880	2.03	UPL4; ubiquitin-protein ligase
AT2G36380	2.03	PDR6; ATPase. coupled to transmembrane movement of substances
AT1G10040	2.04	Alpha/beta-Hydrolases superfamily protein
**AT4G24910**	**2.04**	**Unknown protein**
AT5G25900	2.04	GA3 (GA Requiring 3); ent-kaurene oxidase/ oxygen binding
AT1G74020	2.05	SS2 (Strictosidine Synthase 2); strictosidine synthase
AT4G37180	2.07	Myb family transcription factor
AT4G15210	2.07	BAM5 (Beta-Amylase 5); beta-amylase
AT5G55970	2.07	Zinc finger (C3HC4-type Ring finger) family protein
AT2G02060	2.09	Homeodomain-like superfamily protein
AT3G21710	2.09	Unknown protein
**AT3G59140**	**2.10**	**ATMRP14; ATPase. coupled to transmembrane movement of substances**
AT1G71950	2.10	Proteinase inhibitor. propeptide
AT4G03030	2.10	Kelch repeat-containing F-box family protein
AT5G03560	2.11	Tetratricopeptide repeat (TPR)-like superfamily protein; cation symporter
AT3G58170	2.12	BS14A (BET1P/SFT1P-Like Protein 14A); SNAP receptor
AT4G11600	2.12	ATGPX6 (Gluthatione Peroxydase 6); glutathione peroxidase
AT1G17840	2.14	WBC11 (White -Brown Complex Homolog Protein 11)
AT3G18280	2.16	Bifunctional inhibitor/lipid-transfer protein
AT4G20110	2.16	Vacuolar sorting receptor
AT1G71030	2.16	MYBL2 (Arabidopsis Myb-Like 2); DNA binding / transcription factor
AT1G13280	2.18	AOC4 (Allene Oxide Cyclase 4); allene-oxide cyclase
AT1G54130	2.19	RSH3 (Rela /Spot Homolog 3); GTP diphosphokinase
AT4G34900	2.19	XDH2 (Xanthine Dehydrogenase 2)
AT4G15610	2.21	Uncharacterised protein family
AT2G46370	2.23	JAR1 (Jasmonate Resistant 1); ATP binding / adenylyltransferase/ catalytic
AT5G07440	2.24	GDH2 (Glutamate Dehydrogenase 2); ATP binding / glutamate dehydrogenase [NAD(P)+]
AT5G40382	2.25	Cytochrome-c oxidase
AT5G41340	2.26	UBC4 (Ubiquitin Conjugating Enzyme 4); ubiquitin-protein ligase
AT1G73010	2.26	Phosphate starvation-induced gene 2 (PS2)
AT4G39235	2.27	Unknown protein
**AT4G26580**	**2.27**	**Protein binding / zinc ion binding**
AT5G16840	2.28	BPA1 (Binding partner of ACD11 1); nucleic acid binding
AT3G20080	2.29	CYP705A15; electron carrier/ heme binding / iron ion binding
**AT3G22600**	**2.31**	**Bifunctional inhibitor/lipid-transfer protein**
AT5G62200	2.32	Embryo-specific protein 3. (ATS3)
AT2G44100	2.32	ATGDI1 (Arabidopsis thaliana Guanosine Nucleotide Diphosphate Dissociation Inhibitor 1)
AT4G36400	2.34	(D)-2-hydroxyglutarate dehydrogenase
AT2G45910	2.36	U-box domain-containing protein kinase family protein
AT5G23850	2.37	Unknown protein
AT5G58690	2.37	Phosphoinositide-specific phospholipase C family protein
AT1G53470	2.38	MSL4 (Mechanosensitive Channel of small Conductance -Like 4)
AT5G57800	2.38	CER3 (Eceriferum 3); binding / catalytic/ iron ion binding
AT2G23030	2.38	SNRK2.9 (SNF1-Related Protein Kinase 2.9); ATP binding / kinase/ protein kinase
AT3G50830	2.39	COR413-PM2 (Cold OLD-Regulated 413-Plasma Membrane 2)
AT3G28940	2.40	AIG2-like (avirulence induced gene) family protein
AT3G44540	2.41	FAR4 (Fatty Acid Reductase 4); binding / catalytic/ oxidoreductase. acting on the CH-CH group of donors
AT5G02380	2.42	MT2B (Metallothionein 2B); copper ion binding
AT2G37540	2.42	Short-chain dehydrogenase/reductase (SDR) family protein
AT3G17000	2.43	UBC32 (ubiquitin-conjugating enzyme 32); ubiquitin-protein ligase
AT4G16520	2.44	ATG8F (autophagy 8f); microtubule binding
AT4G29190	2.44	Zinc finger (CCCH-type) family protein
AT3G55030	2.48	PGPS2 (phosphatidylglycerolphosphate synthase 2)
AT2G23790	2.49	Unknown protein
AT1G63010	2.49	SPX (SYG1/Pho81/XPR1) domain-containing protein
AT1G52080	2.49	AR791; actin binding
AT2G39780	2.50	RNS2 (Ribonuclease 2); RNA binding / endoribonuclease/ ribonuclease T2
AT1G76520	2.51	Auxin efflux carrier family protein
AT5G01600	2.51	ATFER1; ferric iron binding
AT4G27020	2.53	Unknown protein
AT1G72830	2.53	NF-YA3 (Nuclear Factor Y. Subunit A3); transcription factor
AT1G13090	2.55	CYP71B28; electron carrier/ heme binding / iron ion binding
AT5G03520	2.56	ATRAB8C; GTP binding
AT2G07640	2.56	NAD(P)-binding Rossmann-fold superfamily protein
AT3G48530	2.56	KING1 (SNF1-Related Protein Kinase Regulatory Subunit Gamma 1)

AT3G15990	2.57	SULTR3;4 (Sulfate Transporter 3;4); sulfate transmembrane transporter
AT2G33480	2.57	ANAC041 (Arabidopsis NAC domain containing protein 41)
AT1G09500	2.58	Similar to Eucalyptus gunnii alcohol dehydrogenase of Unknown physiological function
AT1G61800	2.59	GPT2; antiporter/ glucose-6-phosphate transmembrane transporter
AT5G11610	2.59	Exostosin family protein
AT4G21980	2.62	APG8A (Autophagy 8A); APG8 activating enzyme
AT2G19310	2.65	HSP20-like chaperones superfamily protein
AT1G54040	2.65	ESP (Epithio Specifier Protein); enzyme regulator
AT3G19990	2.66	Unknown protein
AT1G52565	2.68	Unknown protein
AT1G24040	2.69	GNAT-type N-acetyltransferase. putative
**AT3G09950**	**2.72**	**Unknown protein**
AT3G11900	2.73	Aromatic and neutral transporter 1 (ANT1)
AT2G32150	2.76	Haloacid dehalogenase-like hydrolase family protein
AT4G30470	2.79	Cinnamoyl-CoA reductase-related
AT5G43780	2.79	APS4; sulfate adenylyltransferase (ATP)
AT2G43500	2.84	RWP-RK domain-containing protein
AT1G21920	2.84	Histone H3 K4-specific methyltransferase SET7/9 family protein
AT1G01650	2.84	Signal peptide peptidase. putative
**AT2G44130**	**2.85**	**Galactose oxidase/kelch repeat superfamily protein**
AT4G35300	2.85	Tonoplast monosaccharide transporter2 (TMT2); carbohydrate transmembrane transporter
AT1G74320	2.85	Choline kinase. putative
**AT1G33030**	**2.87**	**O-methyltransferase family 2 protein**
AT1G75540	2.88	STH2 (Salt Tolerance Homolog 2); transcription factor/ zinc ion binding
AT1G02305	2.89	Cathepsin B-like cysteine protease. putative
AT2G26230	2.90	Uricase / urate oxidase / nodulin 35. putative
AT5G17220	2.90	ATGSTF12 (Arabidopsis thaliana Glutathione S-Transferase PHI 12)
AT1G01240	2.91	Unknown protein
**AT3G11410**	**2.93**	**PP2CA (Arabidopsis thaliana Protein Phosphatase 2CA); protein binding**
AT5G19590	2.94	Unknown protein
AT1G08920	2.96	ESL1. a transporter for monosaccharides; sugar transporter. putative
AT5G08380	2.96	AGAL1 (Alpha-Galactosidase 1); catalytic
AT1G23040	2.97	Hydroxyproline-rich glycoprotein family protein
AT2G30070	3.14	ATKT1 (POTASSIUM TRANSPORTER 1)
AT4G03320	3.18	Tic20-IV (Translocon RANSLOCON at the inner envelope membrane of chloroplasts 20-IV)
AT5G16340	3.19	AMP-binding protein. putative
AT3G22200	3.21	Pollen-pistil incompatibility 2 (POP2); 4-aminobutyrate transaminase
**AT5G13580**	**3.21**	**ABC-2 type transporter family protein**
AT1G78860	3.22	Curculin-like (mannose-binding) lectin family protein
AT3G11150	3.26	2-oxoglutarate (2OG) and Fe(II)-dependent oxygenase superfamily protein
AT5G47830	3.28	Unknown protein
AT4G31310	3.28	Avirulence-responsive protein-related
AT5G12200	3.30	Dihydropyrimidinase / DHPase / dihydropyrimidine amidohydrolase
AT5G66650	3.38	Unknown protein
AT1G28130	3.43	GH3.17; indole-3-acetic acid amido synthetase
**AT4G19760**	**3.46**	**Glycosyl hydrolase family protein with chitinase insertion domain**
AT5G07920	3.46	DGK1 (Diacylglycerol Kinase 1); calcium ion binding
AT1G77510	3.49	ATPDIL1-2 (PDI-LIKE 1-2); protein disulfide isomerase
AT4G21580	3.53	Oxidoreductase. zinc-binding dehydrogenase family protein
AT4G22840	3.54	Bile acid:sodium symporter family protein
AT1G13990	3.62	Unknown protein
AT1G80920	3.67	J8; heat shock protein binding / unfolded protein binding
AT4G15960	3.67	Alpha/beta-Hydrolases superfamily protein
AT3G20820	3.67	Leucine-rich repeat (LRR) family protein
**AT4G26080**	**3.68**	**ABI1 (ABA Insensitive 1); calcium ion binding / protein serine/threonine phosphatase**
AT3G17810	3.77	Dihydroorotate dehydrogenase. putative
AT2G37678	3.82	FHY1 (FAR-Red Elongated Hypocotyl 1); transcription regulator
**AT5G57050**	**3.82**	**ABI2 (ABA Insensitive 2); protein serine/threonine phosphatase**
AT3G52060	3.85	Core-2/I-branching beta-1.6-N-acetylglucosaminyltransferase family protein
AT4G32940	3.87	GAMMA-VPE (Gamma Vacuolar Processing Enzyme); cysteine-type endopeptidase
AT3G12220	3.97	Scpl16 (serine carboxypeptidase-like 16); serine-type carboxypeptidase
AT1G62290	4.00	Saposin-like aspartyl protease family protein
AT5G63800	4.03	MUM2 (Mucilage-Modified 2); beta-galactosidase
**AT1G72770**	**4.05**	**HAB1 (Homology to ABI1); catalytic/ protein serine/threonine phosphatase**
AT2G47890	4.05	Zinc finger (B-box type) family protein
AT3G29575	4.07	AFP3 (ABI five binding protein 3)
AT4G29820	4.10	Component of mRNA cleavage factor. putative
AT1G59860	4.11	17.6 kDa class I heat shock protein (HSP17.6A-CI)
AT3G57540	4.11	Remorin family protein
AT3G15534	4.17	Unknown protein
AT2G23000	4.18	Scpl10 (serine carboxypeptidase-like 10); serine-type carboxypeptidase
AT5G54840	4.18	SGP1; GTP binding
AT4G31330	4.20	Unknown protein
**AT1G51140**	**4.20**	**Basic helix-loop-helix (bHLH) family protein**

**AT1G19650**	**4.23**	**SEC14 cytosolic factor. putative / phosphoglyceride transfer protein. putative**
AT5G54080	4.27	HGO (Homogentisate 1.2-Dioxygenase); homogentisate 1.2-dioxygenase
AT5G63070	4.27	40S ribosomal protein S15. putative
AT4G08870	4.30	Arginase. putative
AT2G46680	4.31	ATHB-7 (Arabidopsis thaliana Homeobox 7); transcription activator
AT1G56300	4.31	Chaperone DnaJ-domain superfamily protein
AT3G20250	4.35	APUM5 (Arabidopsis Pumilio 5); RNA binding / binding
**AT5G47720**	**4.42**	**Acetyl-CoA C-acyltransferase. putative / 3-ketoacyl-CoA thiolase. putative**
AT1G72660	4.51	P-loop containing nucleoside triphosphate hydrolases superfamily protein
AT3G09390	4.58	MT2A (Metallothionein 2A); copper ion binding
**AT5G09930**	**4.69**	**ATGCN2; transporter**
AT4G02360	4.72	Unknown protein
**AT3G61450**	**4.75**	**SYP73 (Syntaxin of Plants 73); protein transporter**
AT2G31110	4.79	TBL-type polysaccharide O-acetyltransferase. putative
AT3G49570	4.87	LSU3 (Response to low Sulfur 3)
AT3G23790	4.90	Acyl activating enzyme 16 (AAE16)
AT3G51000	4.94	Alpha/beta-Hydrolases superfamily protein
AT4G28530	4.95	Anac074 (Arabidopsis NAC domain containing protein 74)
AT1G58180	4.96	Beta carbonic anhydrase 6 (BCA6)
AT4G08770	5.04	Peroxidase superfamily protein
**AT4G11650**	**5.22**	**ATOSM34 (osmotin 34)**
AT5G14450	5.36	GDSL-motif lipase/hydrolase family protein
AT4G37010	5.42	Caltractin. putative / centrin. putative
AT4G33120	5.46	Cyclopropane fatty acid synthase
**AT3G17110**	**5.53**	**Pseudogene. glycine-rich protein**
AT5G23750	5.55	Remorin family protein
AT3G32980	5.62	Peroxidase superfamily protein
AT3G57680	5.81	Peptidase S41 family protein
AT4G18830	5.84	OFP5 (Arabidopsis thaliana Ovate Family AMILY Protein 5)
AT4G33020	5.86	ZIP9; cation transmembrane transporter/ metal ion transmembrane transporter
AT1G09310	5.95	Unknown protein
AT1G72100	5.95	Late embryogenesis abundant domain-containing protein / LEA domain-containing protein
**AT1G17870**	**5.98**	**EGY3 (Ethylene -Dependent Gravitropism -Deficient and Yellow-Green-Like 3)**
AT4G24960	5.99	ATHVA22D; Homologous to a eukaryote specific ABA- and stress-inducible gene
**AT1G53580**	**6.06**	**GLY3 (Glyoxalase II 3); hydrolase/ hydroxyacylglutathione hydrolase**
**AT1G64660**	**6.12**	**ATMGL (Arabidopsis thaliana Methionine Gamma -Lyase)**
AT2G43590	6.13	Chitinase family protein
AT2G33830	6.45	Dormancy/auxin associated family protein
**AT1G45249**	**6.58**	**Abscisic acid responsive elements-binding factor 2 (ABF2)**
**AT3G03170**	**6.63**	**Unknown protein**
AT4G39210	6.70	APL3; glucose-1-phosphate adenylyltransferase
AT1G54100	6.78	ALDH7B4 (Aldehyde Dehydrogenase 7B4); 3-chloroallyl aldehyde dehydrogenase
AT3G03870	7.00	Unknown protein
AT3G57520	7.10	AtSIP2 (Arabidopsis thaliana seed imbibition 2)
AT3G05400	7.31	Sugar transporter. putative
**AT4G12290**	**7.48**	**Amine oxidase/ copper ion binding**
AT5G25450	7.84	Ubiquinol-cytochrome C reductase complex 14 kDa protein. putative
**AT3G13672**	**8.65**	**TRAF-like superfamily protein**
AT4G22870	9.13	Sodium Bile acid symporter family; transporter activity
AT1G21000	9.47	Zinc-binding family protein
**AT2G02990**	**9.93**	**RNS1 (Ribonuclease 1); endoribonuclease/ ribonuclease**
AT1G02470	11.12	Polyketide cyclase/dehydrase and lipid transport superfamily protein
**AT4G12580**	**11.22**	**Unknown protein**
AT1G78780	12.01	Pathogenesis-related family protein
**AT4G33150**	**13.77**	**Lysine-ketoglutarate reductase/saccharopine dehydrogenase bifunctional enzyme**
AT5G15250	14.13	FTSH6 (FTSH Protease 6); ATP-dependent peptidase/ ATPase/ metallopeptidase
AT1G80130	16.26	Tetratricopeptide repeat (TPR)-like superfamily protein
**AT4G02280**	**16.27**	**SUS3 (sucrose synthase 3); UDP-glycosyltransferase/ sucrose synthase**
**AT4G17030**	**16.66**	**ATEXLB1 (Arabidopsis thaliana Expansin-Like B1)**
**AT5G10930**	**17.09**	**CIPK5 (CBL-Interacting Protein Kinase 5); ATP binding / kinase**
AT5G59310	17.31	LTP4 (Lipid Transfer Protein 4); lipid binding
AT4G33110	17.64	S-adenosyl-L-methionine-dependent methyltransferases superfamily protein
AT4G30050	17.99	Unknown protein
AT3G03470	23.87	CYP89A9; electron carrier/ heme binding / iron ion binding
AT3G08860	25.32	Pyrimidine 4 PYR4
AT5G45690	25.84	Protein of Unknown function
**AT2G47770**	**27.00**	**TspO-like stress sensory protein**
AT5G59330	66.37	Bifunctional inhibitor/lipid-transfer protein

Additional file 12.

Gene list of the hormone metabolism biological process group in roots or shoots of *H. incana*.

	AGI	FC	Subclasses	Description
**In roots**	At1g45249	5.19	Abscisic acid	Abscisic acid responsive elements-binding factor 2
	At2g47770	9.62		Outer membrane tryptophan-rich sensory protein-related
	At3g02480	23.99		Late embryogenesis abundant protein (LEA) family protein
	At4g18350	2.69		Nine-cis-epoxycarotenoid dioxygenase 2
	At1g30100	5.21		Nine-cis-epoxycarotenoid dioxygenase 5
	At4g26080	3.90		Protein phosphatase 2C family protein
	At5g57050	4.38		Protein phosphatase 2C family protein
	At5g55250	2.35	Auxin	IAA carboxylmethyltransferase 1
	At4g27260	0.39		Auxin-responsive GH3 family protein
	At5g13370	2.59		Auxin-responsive GH3 family protein
	At5g16530	0.44		Auxin efflux carrier family protein
	At5g16010	0.44	Brassinosteroid	3-oxo-5-alpha-steroid 4-dehydrogenase family protein
	At1g77330	0.45	Ethylene	2-oxoglutarate (2OG) and Fe(II)-dependent oxygenase s protein
	At1g62380	3.14		ACC oxidase 2
	At1g52820	0.07	Gibberellin	2-oxoglutarate (2OG) and Fe(II)-dependent oxygenase protein
	At1g52050	0.10	Jasmonate	Mannose-binding lectin superfamily protein
**In shoots**	At4g24960	5.99	Abscisic acid	HVA22 homologue D
	At1g45249	6.58		Abscisic acid responsive elements-binding factor 2
	At2g47770	27.01		Outer membrane tryptophan-rich sensory protein)-related
	At4g26080	3.68		Protein phosphatase 2C family protein
	At5g57050	3.82		Protein phosphatase 2C family protein
	At2g46370	2.23	Auxin	Auxin-responsive GH3 family protein
	At1g28130	3.43		Auxin-responsive GH3 family protein
	At2g33830	3.45		Dormancy/auxin associated family protein
	At2g03760	0.25	Brassinosteroid	Sulphotransferase 12
	At4g30610	0.21		Alpha/beta-Hydrolases superfamily protein
	At5g25190	0.36	Ethylene	Integrase-type DNA-binding superfamily protein
	At1g74670	0.14	Gibberellin	Gibberellin-regulated family protein
	At3g10185	0.41		Gibberellin-regulated family protein
	At5g25900	2.04		Gibberellin-requiring 3
	At1g54040	2.65	Jasmonate	Epithiospecifier protein
	At1g13280	2.18		Allene oxide cyclase 4
