# Supplementary material for: Transcriptome Changes in Hirschfeldia incana in Response to Lead Exposure
Source: Front Plant Sci. 2016 Jan 13;6:1231. doi: 10.3389/fpls.2015.01231 (PMC4710698; doi:10.3389/fpls.2015.01231)
Supplement: Supplementary file 2 [file Data_Sheet_2.PDF]

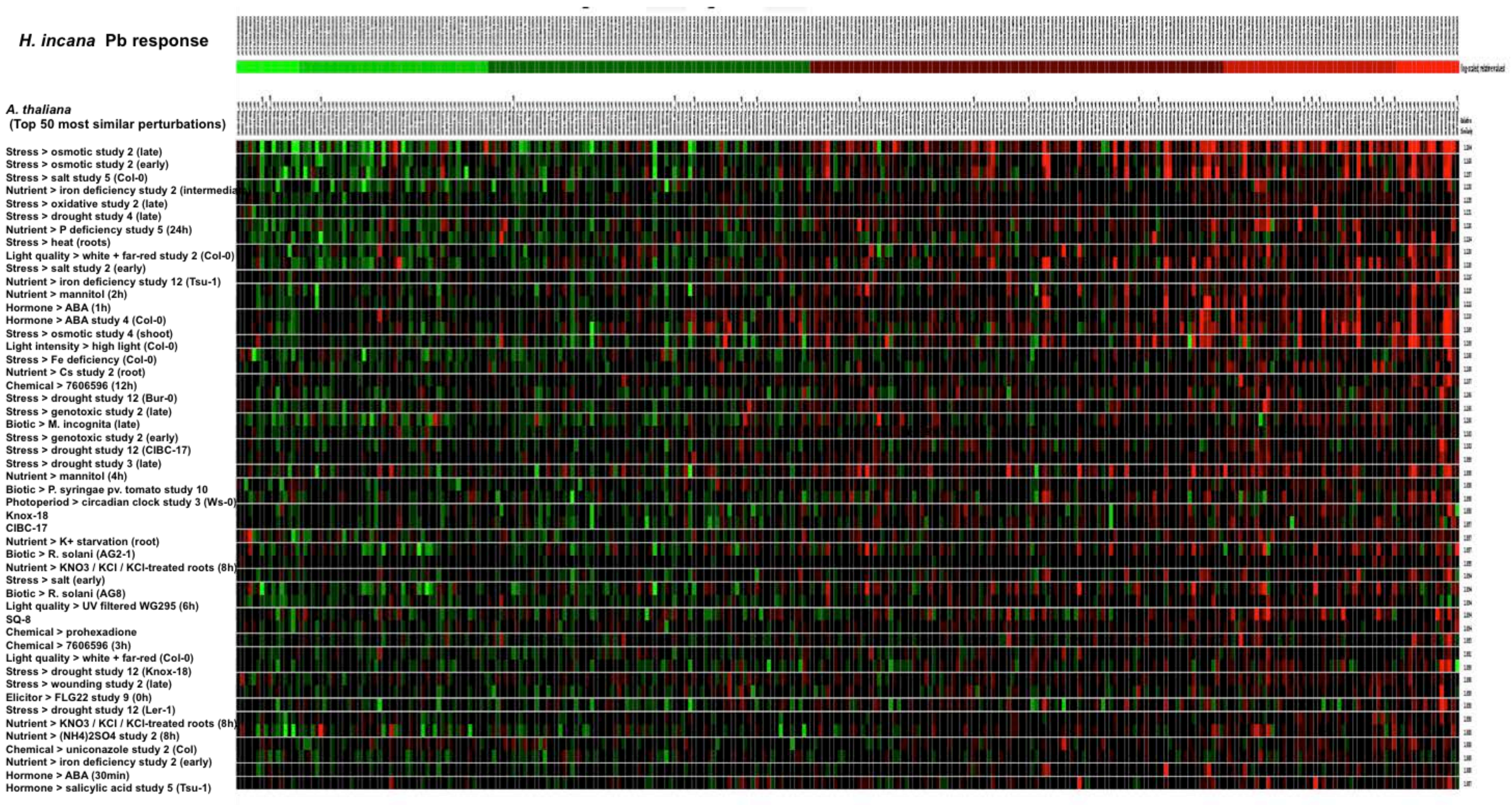

Additional file 10.
Signature analysis between the *H. incana* Pb responsive profile in roots and transcriptome studies on various abiotic stress perturbations from the Genevestigator database.

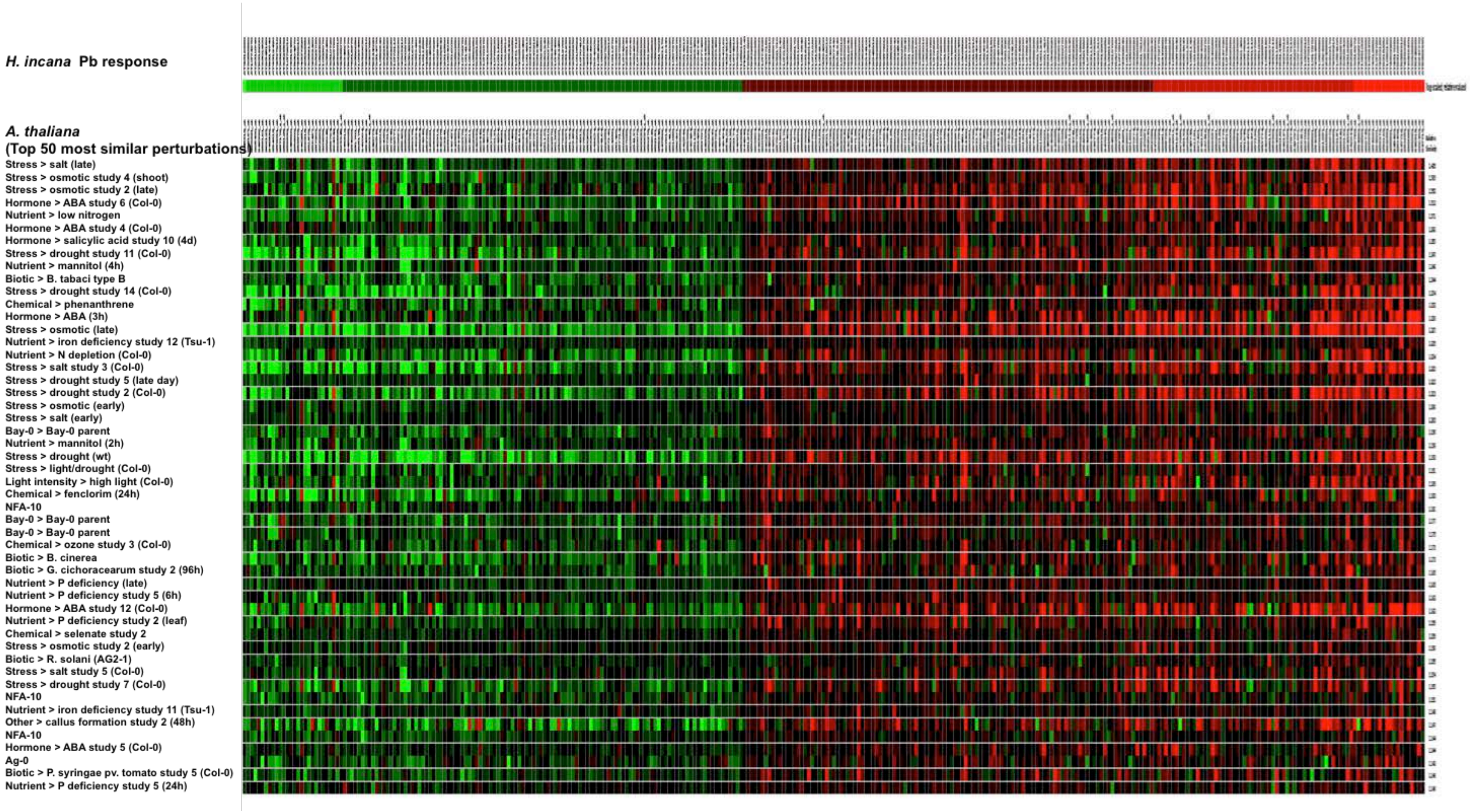

Additional file 11.
Signature analysis between the *H. incana* Pb responsive profile in shoots and transcriptome studies on various abiotic stress perturbations from the Genevestigator database.
